# Factors Affecting Adherence to a Low Phenylalanine Diet in Patients with Phenylketonuria: A Systematic Review

**DOI:** 10.3390/nu16183119

**Published:** 2024-09-15

**Authors:** Roza Yagudina, Andrey Kulikov, Vyacheslav Serpik, Marina Protsenko, Kirill Kopeyka

**Affiliations:** Department of Organization of Medical Provision and Pharmacoeconomics, Sechenov First Moscow State Medical University (Sechenov University), Trubetskaya Str. 8/2, 119991 Moscow, Russia; yagudina_r_i@staff.sechenov.ru (R.Y.); kulikov_a_yu@staff.sechenov.ru (A.K.); serpik_v_g@staff.sechenov.ru (V.S.); protsenko_m_v@staff.sechenov.ru (M.P.)

**Keywords:** phenylketonuria, PKU, dietary adherence, low-Phe diet, compliance, barriers, facilitators, systematic review

## Abstract

Phenylketonuria (PKU) is an inherited metabolic disorder that requires lifelong adherence to a low-phenylalanine (Phe) diet to prevent severe neurological complications. However, maintaining dietary adherence can be challenging for patients and their families. This systematic review aimed to comprehensively evaluate the factors affecting adherence to a low-Phe diet in patients with PKU. A systematic search of multiple databases was conducted, and 49 studies were included in the final analysis. The quality of evidence was assessed using the Joanna Briggs Institute levels of evidence and the Quality Assessment with Diverse Studies tool. The review identified four main categories of factors influencing dietary adherence: family-related factors (social, psychological, behavioral, and educational), patient-specific factors (psychological, behavioral, educational, and demographic), environmental factors (healthcare professional support, educational and camp-based interventions, and the COVID-19 pandemic), and therapy-related factors (protein substitute formulation, clinic visits, blood tests, and telemedicine). The findings highlight the complex interplay between elements contributing to dietary adherence in PKU patients and underscore the importance of a multifaceted approach to support patients and their families. Future research should prioritize high-quality longitudinal and experimental studies to provide stronger evidence for the PKU community.

## 1. Introduction

Phenylketonuria (PKU) is an inborn error of metabolism caused by a deficiency in the enzyme phenylalanine hydroxylase (PAH), which is responsible for converting the amino acid phenylalanine (Phe) into tyrosine [1]. The resulting accumulation of Phe in the blood can lead to severe intellectual disability, seizures, and other neurological problems if left untreated [2]. The primary treatment for PKU is a lifelong low-Phe diet, which involves restricting the intake of high-protein foods and supplementing patients with Phe-free or low-Phe amino acid mixtures [3]. Adherence to this strict dietary regimen is crucial for optimal health outcomes, but it can be challenging for patients and their families [4]. Dietary adherence in PKU is a complex behavior influenced by a multitude of factors, including individual patient characteristics, family dynamics, social support, and healthcare system factors [5]. Understanding these factors is essential for developing targeted interventions to improve adherence and, ultimately, health outcomes for patients with PKU. Previous studies have identified several barriers to dietary adherence, such as the restrictive nature of the diet, a lack of social support, and limited access to specialized care [6,7]. However, a comprehensive understanding of the factors influencing adherence across different age groups and settings is lacking.

Systematic reviews are a valuable tool for synthesizing evidence from multiple studies and identifying gaps in the literature [8]. By systematically searching, appraising, and synthesizing the available evidence, systematic reviews can provide a more reliable and comprehensive understanding of a topic than individual studies [9]. In the context of dietary adherence in PKU, a systematic review can help us to identify the most important factors influencing adherence and inform the development of evidence-based interventions to improve adherence and health outcomes. 

Previous research has identified the influence of demographic and psychosocial factors on treatment adherence in children and adolescents with PKU [10]. While this review provided valuable insights into the factors affecting dietary adherence in pediatric population, there is a need for a comprehensive and up-to-date systematic review on the factors affecting dietary adherence in PKU across all age groups and settings to synthesize the available evidence and identify consistent patterns across studies. 

## 2. Materials and Methods

### 2.1. Search Strategy

The search process used in this systematic review was carried out in accordance with the Preferred Reporting Items for Systematic Reviews and Meta-Analyses (PRISMA) guidelines [11]. The review protocol was registered in the International Prospective Register of Systematic Reviews (PROSPERO registration ID number CRD42023412538) [12]. A systematic search of MEDLINE/PubMed, The Cochrane Library, lens.org, and dimensions.ai databases was performed in November 2023. Abstracts of articles containing data on the prevalence of and/or factors associated with dietary adherence in patients with PKU were identified using keywords with search terms including “phenylketonuria”, “hyperPhemia”, “Phe hydroxylase deficiency”, “adherence”, and “low-Phe diet”, as well as their synonyms and combinations. Details of the search strategy are available in Table S1.

### 2.2. Eligibility Criteria

Eligibility criteria were developed in accordance with the Population, Intervention, Comparison, Outcomes and Study (PICOS) statement [13]. 

Population: Patients with PKU diagnosed according to well-defined criteria. Intervention: Low-Phe diet in patients with PKU.

Comparator: Comparisons were made between adherent and non-adherent patients with PKU to identify factors that influence dietary adherence. 

Outcomes: Studies included a statistical examination of the correlation between therapy-related, socio-demographic, disease-related, and/or psychosocial factors and adherence to the low-Phe diet. For the prevalence of adherence to the low-Phe diet in patients with PKU, adherence was measured by self-report (or parent report), dietitian assessment, physician assessment, or serological markers. Additional outcomes: The documentation of other factors associated with adherence to the low-Phe diet (e.g., environmental factors, family-related factors, patient-specific factors).

Types of studies included: We included qualitative, cross-sectional, case–control, correlational, cohort, randomized controlled trial, and mixed-methods studies available in full-text form and published in English. Letters to the editor, commentaries, abstracts, Editorials, studies centered on animals, systematic reviews, and case reports were excluded.

### 2.3. Study Selection and Data Extraction

Two researchers (KK, VS) independently performed title and abstract screening to identify potentially relevant studies. Full-text articles were then assessed for eligibility based on the predetermined inclusion and exclusion criteria. Any disagreements were resolved through discussion and consensus.

Data extraction was conducted using a standardized form, capturing key information from each included study. The extracted data included the authors, year of publication, article title, study design, participant characteristics (age and sex), sample size, methods for assessing adherence to the low-Phe diet, factors associated with dietary adherence, key findings, and relevant statistical information. Summary tables were created to present the main features and findings of the included studies.

### 2.4. Quality Assessment

To ensure the validity and integrity of the findings of this systematic review, a rigorous quality assessment process was undertaken. Quality assessment was completed independently by two researchers (KK, VS), and conflicts were resolved through consensus. The Joanna Briggs Institute (JBI) levels of evidence (LOEs) were employed to rate the quality of evidence of the included studies [14]. The JBI LOE categorizes studies based on their methodological rigor and susceptibility to bias, with Level 1 representing the highest quality of evidence and Level 5 the lowest.

The JBI levels of evidence hierarchy encompasses the following:

Level 1: Experimental designs, including systematic reviews of randomized controlled trials (RCTs) and other experimental studies;

Level 2: Quasi-experimental designs, such as prospectively controlled studies without randomization;

Level 3: Observational analytic designs, which includes cohort and case–control studies;

Level 4: Observational descriptive studies, such as cross-sectional and case series studies;

Level 5: Expert opinion and bench research, including consensus and single expert opinions.

For the purpose of this review, each included study was classified according to the JBI LOE, and its methodological quality and risk of bias were further evaluated using the Quality Assessment with Diverse Studies (QuADS) developed by Harrison et al. (2021) [15]. The QuADS tool is well validated for assessing research of various designs, and it demonstrates good reliability in differentiating between high- and low-quality studies.

The assessment with QuADS covered 13 key items relevant to the qualitative and quantitative aspects of the studies. Each item was evaluated on a 4-point Likert scale, ranging from ‘not at all’ (0) to ‘complete’ (3). The items addressed critical aspects of study design, including the clarity of aims, appropriateness of methodology, use of robust data collection methods, and relevance of analyses. The scores for each item were summed to provide a total quality score for each study, with a maximum possible score of 39. This quantitative scoring enabled a standardized assessment of study quality, allowing for a clear comparison across studies with diverse methodologies.

### 2.5. Data Synthesis

A narrative synthesis approach was employed to summarize the results reported by each study. This involved identifying commonalities and differences among the studies, evaluating the methodologies used, synthesizing the main findings, and assessing the quality of evidence. Due to the heterogeneity in study populations, methods, and reported outcomes, a meta-analysis was not deemed appropriate. The narrative synthesis allowed for a comprehensive overview of the current evidence on factors affecting adherence to a low-Phe diet in patients with PKU.

## 3. Results

### 3.1. Study Selection

**Figure 1:**
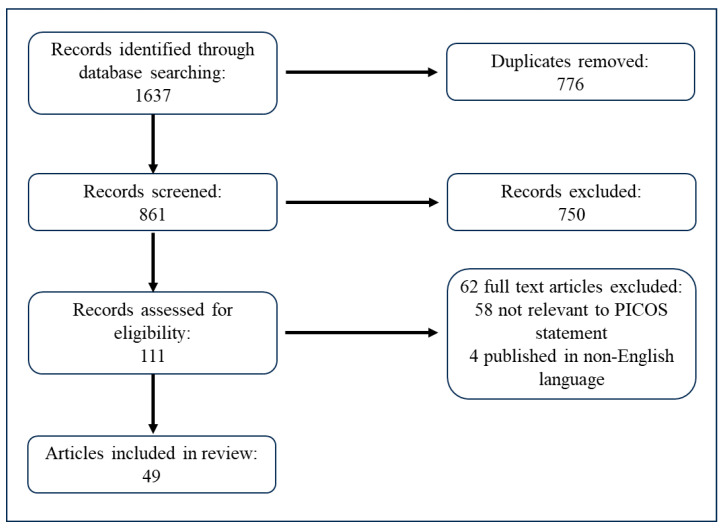
A flow diagram illustrating the search and selection process.

Our comprehensive search across multiple databases initially identified a total of 1637 articles. The screening of titles and abstracts led to the exclusion of 776 duplicates. After removing duplicates, the remaining 861 articles were subjected to a thorough review of titles and abstracts, which resulted in the further exclusion of 750 articles deemed irrelevant for the current review. Consequently, 111 full-text articles were assessed for their eligibility based on the predetermined inclusion and exclusion criteria. Of these, 62 articles were excluded for not meeting the criteria, leaving 49 studies that were included in the final analysis. These studies were evaluated for their exploration of various factors affecting adherence to a low-Phe diet in patients with PKU. The selection process is summarized in a flow diagram provided in Figure 1.

### 3.2. Quality and Risk of Bias Assessment

The systematic review included a diverse array of study designs, as evidenced by the JBI levels of evidence score attributed to each study. Based on the JBI levels, the quality of evidence was varied, ranging from randomized controlled trials (RCTs) to observational studies without control groups. 

Out of the 49 studies included in the review, 3 studies were categorized as Level 1.c, 2 studies were classified as Level 2.c, and studies were classified 2 as Level 2.d. Level 3 studies, which included observational analytic designs, represented the largest proportion of the included studies. This category encompassed 17 studies classified as Level 3.e (observational study without a control group) and 5 studies classified as Level 3.c (cohort study with control group). These studies contribute to the body of evidence but are subject to more bias compared to experimental studies due to the lack of randomization and control. The review also encompassed a significant number of descriptive studies, with 17 studies falling into Level 4.b (cross-sectional study) and 3 studies classified as Level 4.c (case series). These studies offer insights into associations between variables but do not provide a strong basis for causality due to their observational nature and potential confounding factors.

The quality of studies and risk of bias was further evaluated using the QuADS tool, which includes 13 criteria weighted toward methodological quality. The QuADS tool demonstrated good reliability and validity in assessing the risk of bias and quality across diverse study designs. The QuADS assessment revealed a range of methodological quality among the included studies, with total scores ranging from 18 to 34 out of a maximum of 39 (Table S2) [16,17,18,19,20,21,22,23,24,25,26,27,28,29,30,31,32,33,34,35,36,37,38,39,40,41,42,43,44,45,46,47,48,49,50,51,52,53,54,55,56,57,58,59,60,61,62,63,64]. On average, studies had a quality score of 27.76 out of 39 across the 13 QuADS items. The quality appraisal found that all of the studies scored high (2–3) in several key areas, indicating a lower risk of bias due to the clear description of the research setting and target population, the appropriateness of the study design to address the stated research aims, the format and content of the data collection tool being appropriate to address the stated research aims, and the method of analysis being appropriate to achieve the research aims. However, studies consistently scored lower (0–1), suggesting a higher risk of bias in other important methodological areas, such as the consideration and involvement of research stakeholders in study design or conduct (82%), evidence of consideration of the sampling approach (55%), the justification for the choice of analytic method (33%), the provision of recruitment data (29%), and critical discussion of study strengths and limitations (24%).

### 3.3. Description of Included Studies

Table 1 shows the overview of the studies included in the systematic review, encompassing details such as the study year, the number of participants, participants’ sex, the ages(s) of participants, the level of evidence, and the study design.

The studies were categorized based on the age groups of participants and the primary focus of the research as follows:

1. Children and adolescents (<18 years): Studies that primarily involve children or adolescent participants or focus on issues specific to this age group;
2. Adults (>20 years): These studies exclusively involve adult participants or focus on issues specific to adults with PKU;
3. Mixed age groups: Studies that include participants from various age groups or do not specify a particular age range, covering a broad spectrum of PKU patients;
4. Caregivers/Parents: These studies focus on the roles of caregivers or parents in managing PKU, their knowledge, and its impact on patient outcomes.

This categorization allows for a distinction between studies focusing on specific age groups while also recognizing the importance of caregiver/parent involvement and studies that span multiple age groups.

Our comprehensive review, encompassing a diverse range of studies spanning several decades and involving patients of all ages, caregivers, and healthcare providers, identified four main categories of factors influencing adherence to a low-Phe diet in patients with PKU: family-related factors, patient-specific factors, environmental factors, and therapy-related factors. It is important to note that the dietary treatment of PKU has evolved significantly over the years, particularly since 2000, with improvements in protein substitutes and the increased availability of low-protein foods. While we included pre-2000 studies, we carefully considered their context and relevance. These earlier studies primarily focused on psychosocial, family-related, and patient-specific factors rather than therapy-related aspects. As these factors remain relevant to adherence regardless of changes in dietary treatment, we deemed their inclusion valuable. This approach allowed us to leverage historical insights on enduring psychosocial factors while maintaining awareness of the evolving nature of PKU dietary management.

### 3.4. Family-Related Factors

We recognize that the family constitutes the primary support system for individuals managing PKU, and the dynamics within this unit can either facilitate or hinder dietary compliance. Family-related factors encompass a range of social, psychological, and behavioral components within the family unit that collectively influence dietary compliance. An overview of the research discussed in this section is presented in Table 2.

#### 3.4.1. Social Factors

Several social factors related to family structure and dynamics have been associated with adherence to a low-Phe diet in patients with PKU. 

Family structure plays a role in dietary adherence, with compliant families of both high (M = 55.63, SD = 13.50) and low (M = 57.87, SD = 9.01) socioeconomic statuses having a more structured family environment and more firmly fixed rules than noncompliant families of high (M = 44.25, SD = 8.06) and low (M = 52.00, SD = 10.42) socioeconomic statuses [16].

Family size has been investigated in relation to dietary adherence, although the findings are mixed. One study found no significant relationship between family size and dietary adherence, with blood Phe concentrations not increasing significantly with an increase in family members [41]. However, another study found that among patients who lived with only one parent (n = 17/55, 30.9%), 15 (27.3%) were non-adherent (*p* = 0.027), and adjusted analysis revealed that living with both parents is a protective factor for treatment adherence (RRs 0.59, 95% CI 0.39–0.80, *p* = 0.001) [44].

Parental employment status has also been associated with dietary adherence. One study found a significant association between unemployed parents and higher levels of blood Phe concentration (*p* = 0.03) [41]. Although specific “Phe free” formulas are given at no cost to families in some referral hospitals, unemployed families may be under pressure to pay for other requirements for their children. A significant positive correlation was found between the number of affected children in the family and blood Phe concentrations (r = 0.43, *p* < 0.001), suggesting that an increase in affected children may lead to more financial pressure on families, as special foods or supplements are not always available free of charge [41].

Patients with separated or divorced parents were more likely to have higher Phe levels, and this association was robust and not diminished by adjustment for potential confounding factors [34]. Among patients with parents who were still together, the statement “I drink amino acids 3–4 times/day” was statistically significantly associated with the recommended Phe level (adjusted odds ratio of 0.07, 95% CI 0.006 to 0.783, *p* = 0.031). Another study found a significant association between divorced parents and higher levels of blood Phe concentration (*p* = 0.02), with the mean plasma Phe concentration in patients with divorced and not divorced parents being 14.56 mg/dL and 7.86 mg/dL, respectively [41].

The mother’s country of birth impacts children’s Phe levels in PKU management, with foreign-born mothers’ children typically showing higher Phe concentrations. This is likely due to language barriers, cultural dietary differences, and varying adherence to medical instructions [30]. Healthcare workers often struggle to provide effective dietary advice across cultures due to limited knowledge of diverse eating habits and ingredients [30]. To improve PKU management in diverse populations, culturally sensitive approaches, including translated materials, cultural mediators, and education on various dietary practices, are essential.

#### 3.4.2. Psychological and Behavioral Factors

Parental attitudes and behaviors have been shown to significantly impact adherence to a low-Phe diet in patients with PKU. A study found strong correlations between Phe levels and the external locus of control for both behavioral dysregulation (r = 0.69, *p* < 0.001) and academic difficulties (r = 0.52, *p* < 0.001) [29]. Children exhibited a similar relationship between Phe levels and the external locus of control for behavioral dysregulation (r = 9.61, *p* < 0.001) [29]. In the context of PKU management, individuals with a higher external locus of control tend to believe that factors outside their personal influence predominantly shape their circumstances. Consequently, these individuals are more likely to attribute elevated Phe levels and the resulting behavioral or academic challenges to external factors beyond their control. Such external factors may include chance occurrences, predetermined fate, or the actions of others, rather than recognizing the direct impact of their own dietary choices and adherence to the prescribed low-Phe diet. This perspective can potentially hinder effective self-management of PKU, as it may lead to a reduced sense of personal responsibility for maintaining optimal Phe levels through dietary compliance. Notably, mothers were found to be more disturbed by behavioral dysregulation than fathers [29], which may influence family dynamics in managing PKU and adhering to dietary restrictions.

Parents’ problem-solving skills were related to their children’s level of disease control [16]. On verbal measures of problem-solving, which involved parents verbally responding to hypothetical disease management scenarios, the quality of response varied with the level of dietary control (*p* < 0.001), with parents of children with good dietary control producing a higher quality verbal response (M = 1.99, SD = 0.24) than noncompliant parents [16]. The number of verbal alternatives suggested also varied with the level of dietary control (*p* < 0.01), with parents of children with good dietary control giving responses that specified a greater number of behaviors in response to each situation (M = 1.38, SD = 0.20) compared to mothers of children with poor dietary control (M = 1.24, SD = 0.11) [16].

Parental wellbeing and family support play crucial roles in a child’s adherence to a low-phenylalanine diet. Medford et al. [45] found that including factors such as perceived family support, parental wellbeing, and the child’s level of dependency in their regression model more than doubled the variance in adherence from 14.7% to 34.1%. This substantial increase highlights the critical importance of family dynamics in successful dietary management. Interestingly, Borghi et al. [56] reported that optimal diet adherence was associated with some unexpected parental characteristics: lower social functioning, greater control of anger expression, and more somatic depressive symptoms. Conversely, parents who tended to express anger outwardly and experience higher mental stress were more likely to have children with elevated Phe levels, indicating poorer adherence. These findings suggest a complex relationship between parents’ psychological factors and their children’s dietary compliance in PKU management.

A study revealed a significant association between caregiver experiences with dietary management and children’s Phe levels in PKU [31]. Children exhibited notably lower Phe levels when their caregivers reported less frequent, less challenging, and less emotionally distressing dietary problems. Moreover, these caregivers perceived their strategies for addressing dietary issues as more effective. All of these associations were statistically significant (*p* values < 0.05) [31]. Caregivers who reported using strategies coded as representing an authoritarian parenting style to solve dietary problems were significantly more likely to have older children with higher Phe levels (all *p* values < 0.05) [31]. The number of formula-related and total problems reported by caregivers was significantly related to children’s Phe levels (r = 0.51, *p* = 0.03 and r = 0.47, *p* = 0.05, respectively), with more problems being related to worse adherence [31]. Caregivers’ perceptions of the effectiveness of dietary solutions were significantly related to their children’s Phe levels (r = 0.64, *p* < 0.01), with greater effectiveness ratings associated with better adherence levels [31]. Children who reported trying strategies coded as being maladaptive for adherence (i.e., eating inappropriate foods or ignoring/avoiding problems) had significantly higher Phe levels, i.e., F(1, 8) = 8.38, *p* < 0.05 [31].

#### 3.4.3. Educational Factors

Higher parent education levels were associated with lower blood Phe levels, with significant correlations found between blood Phe and maternal education (r = −0.27, *p* < 0.05) and paternal education (r = -0.28, *p* < 0.05) [17]. However, a multiple logistic regression analysis did not find a statistically significant association between parental educational level and Phe levels [34]. When considering the quantity or excess of Phe intake compared to the prescribed diet, children with mothers with a lower educational level showed a significant excess (median 158 mg vs. −36 mg, *p* = 0.045), while no difference was found based on fathers’ education levels (median −35.5 vs. −2.00, *p* = 0.9) [40]. Additionally, patients whose mothers had 4 years of formal education or less were at a greater risk of non-adherence (RR 1.59, 95% CI 1.01–2.51, *p* = 0.044) [44].

Parental disease-related knowledge levels also play a role in adherence to a low-Phe diet. A study evaluating the success of a PKU education program found that subjects who successfully completed the program had a lower baseline blood Phe concentration (13.4 mg/dL) compared to nonsuccessful subjects (17.9 mg/dL, *p* < 0.05) [18]. A significant relationship (r = −0.485) was found between baseline blood Phe concentration and success with the program [18].

Linear regression analysis showed that greater knowledge was associated with lower Phe concentrations, but this association was statistically significant only in the parent group (parents: b = −28, 95% CI −39/−17; patients: b = −20, 95% CI −42/2) [27]. However, after adjusting for confounders (pre-treatment Phe concentrations, dietary Phe tolerance, patient age, parental educational level, and ethnicity), this association disappeared (parents: b = 0.4, 95% CI -8/7; patients: b = 1.5, 95% CI -10/7) [27]. A negative correlation was found between maternal knowledge about Phe exchange and median blood Phe concentration in the child (*p* < 0.05) [36]. “Phe exchange”, or “PKU exchange”, is a dietary management tool used for individuals with phenylketonuria (PKU). It refers to a measured amount of Phe in food. One standard exchange is defined as 15 mg of Phe. This system allows patients and caregivers to categorize and measure foods in terms of these exchanges, facilitating precise tracking and control of Phe intake. Maternal knowledge about a standard 15 mg Phe exchange system was correlated with dietary compliance, as measured by blood Phe concentrations [36].

### 3.5. Patient-Specific Factors

We identified three main categories of patient-specific factors that may influence adherence to a low-Phe diet in patients with PKU: psychological and behavioral factors, educational factors, and demographic factors. The key findings from the studies explored in this section are summarized in Table 3.

#### 3.5.1. Psychological and Behavioral Factors

In a pre-2000 study, Waisbren et al. (1997) found that adult women (>18 years) who felt that they did not receive support from health professionals needed extensive help from their parents and believed that life’s circumstances were determined through chance (an external locus of control) were at greater risk of poor metabolic control [20]. 

Improved attitudes and reduced negative health beliefs among children and adolescents were not accompanied by sustained reductions in blood Phe levels [24]. Believing that the costs of treatment add to the complications of the diet was associated with late metabolic control (RR, 4.2; 95% CI, 1.4, 23.3) [25]. Other factors associated with increased risk were age (<25 years) (RR, 8.4; 95% CI, 1.9, 147.8), having a high school education or less, and dependence on state assistance programs [25].

Significant correlations were found between Phe levels and the external locus of control for academic difficulties (*p* < 0.001) and between Phe levels and parents’ emotional responses to their children’s behavior/academic difficulties (*p* < 0.001) [29]. Children’s perceptions of the effectiveness of strategies were significantly associated with their Phe levels for dietary problems (*p* < 0.05) [31]. Children who reported using maladaptive strategies (e.g., eating inappropriate foods or ignoring/avoiding problems) had significantly higher Phe levels, *p* < 0.05 [31].

Among adult PKU patients, full-time work was associated with poorer metabolic compliance (mean Phe blood levels > 281.11 μMol/L) compared to part-time work. Shift work was linked to even worse compliance, with mean Phe plasma levels exceeding 356.73 μMol/L [47]. Perceived barriers related to PKU treatment also affected adherence. Adolescents reported fewer barriers compared to adults (U = 8.000, *p* = 0.008), and patients with better recent metabolic control reported fewer perceived barriers than patients with poor adherence (U = 20.000, *p* = 0.009) [53]. The number of perceived barriers was positively associated with recent blood Phe concentration (Kendall’s tau(b) = 0.41; *p* = 0.001) [53].

#### 3.5.2. Educational Factors

Patients’ education level has been shown to influence adherence to a low-Phe diet in patients with PKU. Brown et al. (2002) found that among adults (>18 years), having a high school education or less was associated with an increased risk of late metabolic control (RR, 8.4; 95% CI, 1.9, 147.8) [25]. Additionally, patients who achieved a university degree were found to adhere to the PKU special diet throughout their lives, as they understood the reasons for being on a special diet and thus avoided any damage to their psychomotor functions [50]. Patients’ disease-related knowledge also plays a crucial role in adherence to a low-Phe diet. Children and adolescents (<18 years) who successfully completed an educational program had a significantly lower baseline blood Phe concentration (13.4 mg/dL) compared to nonsuccessful subjects (17.9 mg/dL) (*p* < 0.05) [18]. However, increased knowledge, improved attitudes, and reduced negative health beliefs were not always accompanied by sustained reductions in blood Phe levels [24,35]. A significant difference in the extent of change in knowledge score between baseline and 1 month was found in favor of the intervention group (*p* < 0.05), but this improvement in knowledge was not accompanied by a significant improvement in measures of compliance [35]. Another study found significant associations between knowledge scores (total knowledge, PKU knowledge, PKU diet knowledge) and whether an adult respondent always followed a PKU diet, had returned to diet, or was currently off diet [64]. Post hoc analysis showed significantly lower correct scores among those who were currently off diet (mean ± SD total knowledge score 69.1% ± 15.4%) compared to those who had always followed their recommended PKU diet (78.0% ± 12.0%, *p* = 0.005) [64].

#### 3.5.3. Demographic Factors

Patient age was found to be a significant factor affecting adherence to a low-Phe diet in patients with PKU across multiple studies involving various age groups [19,21,23,25,30,33,34,39,40,43,45,46,47,48,49,51,54]. Blood Phe concentrations were significantly higher in older age groups compared to younger groups [19,23,39,43,48]. Pre-2000 studies by McMurry et al. (1992) and Schulz and Bremer (1996), both involving mixed age groups, reported that blood Phe concentrations were significantly higher in older age groups compared to younger groups, with age positively correlated with mean Phe concentrations [19,21]. Non-adherence to clinic-recommended target Phe concentrations increased with age [46]. A linear relationship was found between age and mean Phe blood levels, with an average annual increase of 30.56 μMol/L (95% CI: 7.53; 53.60) [47]. Age was the single most robust and statistically significant predictor of Phe concentrations, with a baseline concentration of 140 μmol/L and an increase of 22 μmol/L with every additional year of age (*p* < 0.0001) [49]. Dietary compliance, as measured by questionnaire, was lower in older subjects compared to younger groups and negatively correlated with age (r = −0.689, *p* < 0.0001) [19]. Factors associated with late metabolic control included age < 25 years (RR, 8.4; 95% CI, 1.9, 147.8) [25]. Child age accounted for significant variance in the proportion of blood Phe concentrations in the target range (b = 1.879, *p* = 0.009, r^2^ = 0.147) [45]. Logistic regression analyses identified higher Phe levels in the age ranges of 12–23 months and 6–8 years as predictors of treatment discontinuation before 13 years of age [48].

The impact of gender on adherence to a low-Phe diet in PKU patients was less consistent across studies involving various age groups [23,34,40,42,43,54]. Some studies found no significant difference in blood Phe levels between boys and girls [23,40,43]. However, one study reported that girls had lower Phe levels than boys, with sex differences achieving borderline statistical significance (adjusted odds ratio of 0.004, 95% CI 0.000 to 1.011, *p* = 0.05) [34]. Another study found that the mean proportion of females (all ages, 57.5%) with 70% or more of their Phe concentrations within the target range was higher than that for males (all ages, 49.8%) [42]. In contrast, one study reported that those who had a blood Phe concentration higher than the recommended threshold were more likely to be female (39.5% of females vs. 21.5% of males, *p* = 0.0202) [54]. However, in this study population, females (median = 16.6 years) were older than males (median = 9.6 years) (*p* = 0.0088), which may have skewed the differences in blood Phe concentration [54].

### 3.6. Environmental Factors

Environmental factors encompass a wide range of external influences that can affect a patient’s ability to adhere to a low-Phe diet. An overview of the research discussed in this section is presented in Table 4, which summarizes key studies, their participants, and their main findings related to environmental factors affecting dietary compliance in PKU.

Multiple logistic regression analyses revealed that adult women who felt that they did not receive support from health professionals were at greater risk of poor metabolic control [20]. On the other hand, all adult participants who were either mildly or moderately depressed experienced a beneficial effect on their depression when they were psychologically supported, which was further enforced by the reduction in Phe blood levels after their psychological support [50]. These findings highlight the importance of support from health professionals in promoting adherence to a low-Phe diet and improving metabolic control in patients with PKU.

Educational and camp-based interventions have been shown to improve adherence to a low-Phe diet in patients with PKU [22,24,38,57,62]. A week-long camping experience for girls and young women resulted in enhanced ongoing social support networks and improved metabolic control, with blood Phe concentrations reduced in 96% (24/25) of the young women during camp and remaining down in 75% (18/24) of these women at follow-up (*p* < 0.05) [22]. However, increased knowledge gained from educational interventions was not always accompanied by sustained reductions in blood Phe levels [24]. An educational intervention was found to be more effective at lowering children’s Phe levels compared to a control group (F = 4.68, *p* = 0.03), with the percentage of children in the educational group with a normal range of Phe levels increasing from 26% and 38% at baseline to 73.9% at 24-month follow-up [57]. Additionally, a camp-based intervention for mixed age groups led to decreased plasma Phe concentrations [median change: −173 µmol/L (IQR: −325, −28 µmol/L)], with 70% of PKU participants demonstrating improved dietary adherence by decreasing Phe intake and/or increasing medical food consumption [62].

A study comparing concurrent biochemical markers (Phe, tyrosine, and Phe to tyrosine ratio) at holiday and non-holiday time points found no significant difference in concurrent Phe levels across time using a within-subjects *t*-test (t = 1.029, *p* = 0.331) [37]. Furthermore, no significant differences were observed between Phe levels in 2005 and the two testing occasions, even when adjusted for age and different dietary restrictions. No significant differences were found between lifetime Phe levels and concurrent Phe levels on holiday [t(7) = 1.886, *p* = 0.101] or non-holiday [t(7) = 0.819, *p* = 0.440] occasions [37]. These findings suggest that adherence to a low-Phe diet may not differ significantly between holiday and non-holiday periods.

Several studies involving mixed age groups have investigated the influence of the relationship between the distance to the PKU clinic and clinic staffing resources on adherence to a low-Phe diet in patients with PKU. In one study, no significant correlation was found between the distance in miles and the Phe target (*p* = 0.62) [43]. Similarly, another study reported that the distance between the patient’s town of origin and the PKU clinic did not correlate with median Phe concentration in the 12 months prior to study inclusion, both for adherent patients (*p* = 0.629) and non-adherent patients (*p* = 0.72) [44]. However, clinic staffing resources, defined as the number of specialists per 100 actively managed PKU patients, were found to be correlated with the proportion of non-adherent patients (those not following clinic recommendations for blood Phe concentrations) in different age groups [46]. This suggests that while the physical distance to the clinic may not directly impact adherence, the availability of specialized staff at the clinic could play a role in helping patients to maintain a low-Phe diet.

The COVID-19 pandemic has had varying effects on adherence to a low-Phe diet in PKU patients. One study found that among the child population (GROUP A), metabolic control did not differ significantly during March–April–May (MAM) 2019 compared to MAM 2020. However, adolescent and adult patients (GROUP B) demonstrated a significant decrease in blood Phe concentrations, with significant improvements in metabolic control (556.4 ± 301 μmol/L in MAM 2019 vs. 454 ± 252 μmol/L in MAM 2020) [58]. The authors suggest that this improvement in adherence among adolescent and adult patients may be related to a more favorable social environment with fewer external influences during the pandemic, allowing them to concentrate more on their diet [58]. Another study found that self-reported high stress intensity was associated with higher odds ratios for an increase in Phe concentrations (*p* = 0.0023) and non-PKU-related health problems [59]. Better compliance was associated with higher odds of acceptance of remote contact, reporting fewer problems with contacting the doctor, and lower odds of missing Phe testing. However, a third study reported that the median Phe level significantly increased in both age groups during the COVID-19 era (CE) compared to the non-COVID-19 era (NCE). There was a decreasing tendency in the number of patients within the target Phe range in both age groups during CE [63]. Significant negative correlations were found between the dried blood spot (DBS) testing frequencies and Phe levels in both age groups during NCE (children r = −0.43, *p* = 0.002; adolescents r = −0.37, *p* = 0.012) and in the adolescent group during CE (r = −0.6, *p* = 0.006) [63]. These findings suggest that the COVID-19 pandemic has had mixed effects on adherence to a low-Phe diet in PKU patients, with some studies reporting improvements in adherence, particularly among adolescent and adult patients, while others have reported worsening adherence and increased Phe levels. The impact of the pandemic on adherence may depend on various factors, such as stress levels, access to remote healthcare, and changes in social environments and routines.

### 3.7. Therapy-Related Factors

The management of phenylketonuria (PKU) involves various therapeutic approaches, each with its own set of challenges and potential impacts on dietary compliance. Therapy-related factors include a diverse array of elements, such as the type and formulation of protein substitutes, treatment protocols, the frequency of clinical visits and blood tests, and the use of innovative technologies in patient care. Table 5 synthesizes the key findings from various studies examining the relationship between therapy-related factors and dietary compliance among individuals with PKU.

The formulation of protein substitutes has been shown to impact compliance across different age groups. In a study involving mixed age groups, MacDonald et al. (2003) found that 90% (18 out of 20) of patients took Phe-free amino acid tablets as prescribed compared to only 65% (13 out of 20) who were fully compliant with their usual protein substitute. Additionally, plasma Phe levels were lower when taking tablets, with a median difference of 46 µmol/L (95% CI 14.8 to 89.0, *p* = 0.02), indicating better metabolic control. Similarly, a liquid protein substitute was found to be more popular and efficacious, reducing self-consciousness and improving overall compliance in teenagers and adults with PKU (*p* < 0.0001) [32]. The use of large neutral amino acid (LNAA) supplements was associated with improved adherence, with all patients self-reporting high adherence to medication after 12 months of LNAA treatment, compared to only 3 out of 12 patients reporting ‘medium’ adherence before treatment. Tyrosine levels also increased significantly in 92% of patients (mean 75 ± 16 µmol/L; *p* = 0.0195) [52]. It is important to note that recent research suggests that GMP-based formulas may be more acceptable to patients struggling with dietary adherence when compared to amino acid-based formulas, potentially enhancing compliance and improving health outcomes, especially in adults. [65].

The formulation of the protein substitute appears to impact compliance, with a study showing that 90% of patients took Phe-free amino acid tablets as prescribed, compared to only 65% who were fully compliant with their usual protein substitute. Moreover, plasma Phe levels were significantly lower in patients on the amino acid tablets (*p* = 0.02) [26].

The frequency of visits to a specialist and blood Phe tests was found to be higher in the first year of life compared to subsequent years (visits: OR = 6.8267, 95% CI = 2.827–16.5163, *p* < 0.0001; blood tests: OR = 2.7875, 95% CI = 1.0467–7.4234, *p* < 0.0402) [51]. Phe levels were correlated with the number of visits to a specialist (ρ = 0.39), and the number of Phe blood tests was correlated with the index of dietary control (ρ = −0.33) [51].

The clinic’s Phe target also influenced adherence, with clinics using a higher upper target (600 μM vs. 360 μM) for adult patients having more non-adherent patients when evaluated against a single adherence criterion of 360 μM (*p* < 0.05) [46]. However, adherence to clinic-recommended blood Phe concentrations was not significantly correlated with adherence to blood frequency testing recommendations or actual blood frequency testing, except for patients who were pregnant or planning to become pregnant.

Patients with classical PKU showed higher current blood Phe levels than those with mild PKU (U = 37.000, *p* = 0.003) [55]. Lifetime and childhood Phe levels were associated with recent metabolic control, and the perception of barriers to treatment was associated with higher blood Phe levels.

The implementation of a transition program from pediatric to adult services was found to be successful, with a positive impact on metabolic control and high attendance rates [60]. The mean ± SD of median blood Phe remained stable (525 ± 248 µmol/L vs. 552 ± 225 µmol/L; *p* = 0.100), while the median percentage of blood Phe < 480 µmol/L decreased (*p* = 0.041) [60]. The median number of clinic appointments increased from the first to the second study period (*p* < 0.001) [60].

The treatment approach also played a role in adherence, with children aged 2 to 12 years who received care from an expert, coordinated team in Nova Scotia having better Phe control and more medical visits than those in New Brunswick (*p* < 0.01) [28].

Lastly, the use of telemedicine was associated with a statistically higher ratio of samples with Phe levels in the recommended ranges across all treatment modalities (*p* < 0.05) [61]. In the low-Phe diet group, the decrease in Phe washout frequency and the increase in Phe tolerance were statistically significant during the pandemic (*p* < 0.05) [61].

## 4. Discussion

**Figure 2:**
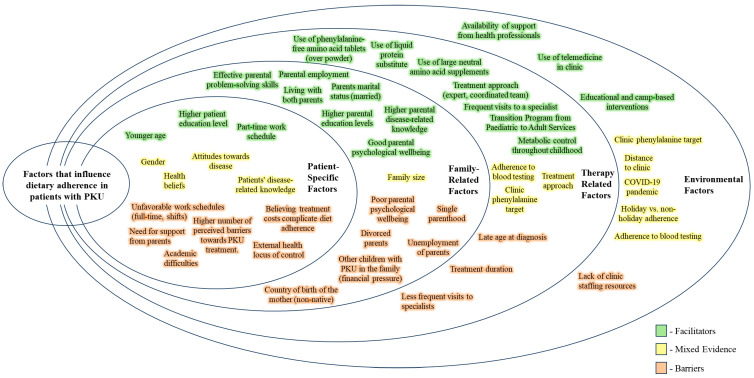
A summary of facilitators and barriers to dietary adherence.

Numerous factors influence dietary adherence in PKU patients, and they can be classified as facilitators, barriers, or mixed evidence based on their impact (Figure 2). 

When considering patient-specific factors, younger age, higher education level, and part-time work schedules are linked to better adherence. Conversely, reliance on parental support, an external health locus of control, the belief that treatment costs complicate diet adherence, academic difficulties, older age, unfavorable work schedules, and a higher number of perceived barriers to PKU treatment are obstacles to adherence.

Family-related factors play a crucial role in promoting adherence, especially for children and adolescents. Effective parental problem-solving skills, parental employment, living with both parents, higher parental education levels, good parental psychological well-being, and better parental disease-related knowledge all contribute positively to adherence. On the other hand, adherence is hindered by poor parental mental health, single parenthood, parental unemployment, having siblings with PKU, parental divorce, and having a mother born outside the country. 

Therapy-related factors such as the formulation of the protein substitute (with tablets or liquid form being preferable), more frequent visits to specialists and blood Phe tests, lower clinic Phe targets, and the implementation of transition programs from pediatric to adult services are associated with better adherence. Classical PKU, higher perceived barriers to treatment, and lower adherence to blood testing frequency remain challenges across all age groups.

Lastly, the availability of support from health professionals, educational and camp-based interventions, and the use of telemedicine in clinics are environmental factors that facilitate adherence, while a lack of clinic staffing resources poses a challenge.

Several limitations should be acknowledged. While the dietary management of PKU has evolved significantly over time, with substantial improvements in protein substitutes and increased availability of low-protein foods, we determined that the inclusion of pre-2000 studies was scientifically justified for several reasons. Pre-2000 studies predominantly explored psychosocial, family-related, and patient-specific factors influencing dietary adherence. These factors, such as family dynamics, patient attitudes, and psychological and social mechanisms underlying adherence, are less likely to be affected by changes in dietary treatment modalities over time. Therefore, insights from earlier studies on these aspects remain relevant to contemporary PKU management. Including earlier studies provides a valuable historical context, allowing for the examination of trends and persistent challenges in PKU management over time. We noted that pre-2000 studies contained minimal data on therapy-related factors that would be outdated by current standards. To address this, we were careful to interpret pre-2000 data within their historical context, particularly when discussing therapy-related aspects. 

PKU is classified as an orphan disease, meaning that it affects a relatively small number of individuals. This inherently limits the potential recruitment pool for studies, often resulting in small sample sizes. Small cohorts can lead to a lack of statistical power to detect meaningful differences or associations.

A considerable number of included studies were categorized as observational or cross-sectional studies. These studies were prone to selection bias and could not control for confounding variables as effectively as randomized controlled trials. The absence of longitudinal or follow-up data in cross-sectional and most observational studies limits the understanding of the long-term effects and sustainability of the outcomes reported. In observational studies without control groups, the inability to control for all potential confounding variables may lead to biased estimates of effect sizes or associations.

The included studies demonstrated strengths in clearly stating their research aims, describing the study context, and selecting appropriate designs; there is significant room for improvement in involving stakeholders, justifying analytic approaches, and transparently discussing study limitations. While this systematic review contributed valuable insights into PKU management, there is a clear need for more high-quality research, particularly longitudinal and experimental studies, that can provide stronger evidence for the PKU community. Future research should prioritize high-quality RCTs and longitudinal studies that can provide more definitive evidence regarding the efficacy of interventions.

## 5. Conclusions

In this systematic review, we have identified a nuanced landscape of influences that span social, psychological, behavioral, educational, and environmental factors influencing dietary adherence in PKU patients across their lifespans. Family-related factors, including parental education, family dynamics, and socioeconomic status, play a significant role in dietary adherence, particularly for children and adolescents. Higher levels of parental education correlate with better dietary control, indicating the need for targeted educational programs for caregivers. Stable family structures and problem-solving abilities further enhance adherence, pointing toward the benefit of psychological and social support services for PKU families. As patients age, the challenges to adherence evolve. Patient-specific factors, such as age and the need for support, also impact adherence, with adolescents and adults facing more significant challenges. This suggests a potential gap in transitional care from pediatric to adult services, highlighting the importance of age-appropriate interventions and support mechanisms to ensure continued adherence throughout a patient’s life. Environmental factors, such as the availability of healthcare support and educational interventions, are essential in facilitating adherence for all age groups. Camp-based programs and telemedicine have demonstrated effectiveness in improving metabolic control, emphasizing the need for accessible and engaging support infrastructures. Therapeutic factors, including treatment modalities and formulations, influence adherence across all age groups. Preferences for Phe-free amino acid tablets and liquid protein substitutes suggest that patient-centered treatment choices can improve adherence.

In conclusion, adherence to a low-Phe diet in PKU is a multifaceted behavior influenced by the interplay of family-related, patient-specific, environmental, and therapy-related factors. The impacts of these factors vary across different age groups, emphasizing the need for a personalized, age-appropriate approach to PKU management. By recognizing and addressing these diverse influences, healthcare providers can develop more effective strategies to improve dietary adherence and, consequently, health outcomes for PKU patients throughout their lives.

## Figures and Tables

**Table 1:** An overview of the included studies.

Study	Year	Number of Participants	Participants’ Sex	Age of Participants	Level of Evidence	Study Design
Children and adolescents (<18 years)						
Fehrenbach and Peterson [16]	1989	32 PKU patients	15 female, 17 male	Ages: 16 months–16 years	3.e	Observational study without a control group
Shulman, Fisch, Zempel, Gadish, and Chang [17]	1991	43 PKU patients	23 female, 20 male	Ages: 2–11 years	4.b	Cross-sectional study
Gleason, Michals, Matalon, Langenberg, and Kamath [18]	1992	16 PKU patients	7 female, 9 male	Ages: 12–19 years	2.d	Pre-test–post-test control group study
Al-Qadreh et al. [23]	1998	48 PKU patients	28 female, 20 male	Ages: 3–15 years	4.b	Cross-sectional study
Singh, Kable, Guerrero, Sullivan, and Elsas [24]	2000	13 adolescent girls with PKU	All female	Mean age = 13 ± 2 years	2.d	Pre-test–post-test or historic/retrospective control group study
Antshel, Brewster, and Waisbren [29]	2004	30 PKU patients	Not specified	Ages: 8–16 years	4.c	Case series
VanZutphen et al. [33]	2007	15 PKU patients	8 female, 7 male	Age range: 8–20 years (mean age = 14.8 years)	4.b	Cross-sectional study
Olsson, Montgomery, and Alm [34]	2007	41 PKU patients	16 female, 25 male	21 patients aged 8–12 years; 20 patients aged 13–19 years	4.b	Cross-sectional study
Sharman, Sullivan, Young, and McGill [37]	2009	16 (10 PKU + 6 controls)	PKU: 2 female, 8 male; Controls: 2 female, 4 male	PKU: mean 14.4 years; Controls: mean 14.0 years	3.c	Cohort study with control group
Peipert, Rohr, Phornphutkul, and Waisbren [38]	2010	18 PKU patients	7 female, 11 male	Ages: 7–15 years	3.e	Observational study without a control group
Vieira et al. [44]	2015	56 PKU patients	25 female, 31 male	Median age: 12 years	4.b	Cross-sectional study
García et al. [48]	2017	75 PKU patients	33 female, 42 male	Ages: 7–13 years	3.e	Observational study without a control group
Walkowiak et al. [51]	2019	57 PKU patients	37 female, 20 male	Ages: 9–20 years	3.e	Observational study without a control group
Zubarioglu et al. [61]	2022	93 PKU patients (38 on BH4 treatment, 16 on BH4+ low-Phe diet, 39 on low-Phe diet)	41 female, 52 male	BH4 treatment group: 5.12 ± 2.8 years (range: 0.9–12.5); BH4+ low-Phe diet group: 5.6 ± 3.15 years (range: 2.6–14.4); Low-Phe diet group: 10.10 ± 7.4 years (range: 2–41)	3.e	Observational study without a control group
Becsei et al. [63]	2022	86 PKU patients	Not specified	Age range: 0–18 years	3.c	Cohort study with control group
Adults (>18 years)						
Waisbren, Hamilton, St James, Shiloh, and Levy [20]	1995	69 women with PKU	All female	Ages: 16–35 years	2.c	Quasi-experimental prospectively controlled study
Brown et al. [25]	2002	24 pregnant women with PKU	All female	≥18 years of age	4.c	Case series
MacDonald et al. [32]	2006	27 PKU patients	15 female, 12 male	Median age = 30 years (range 8–49 years)	1.c	RCT
Riva, Madotto, Turato, Salvatici, and Indovina [47]	2017	20 adults with classic PKU	13 female, 7 male	Mean age = 32.55 (±7.37) years	3.c	Cohort study with control group
Iakovou and Schulpis [50]	2019	110 PKU patients	Not specified	Mean age = 23.5 years	3.e	Observational study without a control group
Burlina, Cazzorla, Massa, Loro, Gueraldi, and Burlina [52]	2020	12 adult PKU patients	7 male, 5 female	Ages: 19–38 years; mean age: 29.6 ± 6.8 years	3.e	Observational study without a control group
Firman, Ramachandran, and Whelan [64]	2022	137 adults with PKU	78 female, 59 male	Ages: 16–65 years; mean age: 34 years and 4 months	4.b	Cross-sectional study
Mixed age groups						
McMurry, Chan, Leonard, and Ernst [19]	1992	26 PKU patients	Not specified	Ages: 1.9–25.5 years	3.e	Observational study without a control group
Schulz and Bremer [21]	1995	99 PKU patients	60 female, 39 male	Ages: 12–29 years	3.e	Observational study without a control group
Waisbren et al. [22]	1997	25 women with PKU	All female	Ages: 11–32 years (median age = 15 years)	3.e	Observational study without a control group
MacDonald et al. [26]	2003	21 PKU patients	13 female, 6 male	Ages: 8–25 years	1.c	RCT
Camfield, Joseph, Hurley, Campbell, and Sanderson [28]	2004	108 PKU patients	Not specified	Median age = 9 years	4.b	Cross-sectional study
Durham-Shearer, Judd, Whelan, and Thomas [35]	2008	32 PKU patients	20 female, 12 male	Ages: 13–42 years	2.c	Quasi-experimental prospectively controlled study
Viau et al. [39]	2011	55 PKU patients	28 female, 27 male	Ages: 6–33 years	3.e	Observational study without a control group
Cotugno, Nicolò, Cappelletti, Goffredo, Dionisi Vici, and Di Ciommo [40]	2011	41 PKU patients	16 female, 25 male	Ages: 3–24 years (median age 8 years)	4.b	Cross-sectional study
Alaei, Asadzadeh-Totonchi, Gachkar, and Farivar [41]	2011	105 PKU patients	59 female, 46 male	Ages: 1–27 years	4.b	Cross-sectional study
Macdonald, Nanuwa, Parkes, Nathan, and Chauhan [42]	2011	125 PKU patients	73 female, 52 male	20 adults (18+ years) and 105 children (up to age 17)	3.e	Observational study without a control group
Freehauf, Van Hove, Gao, Bernstein, and Thomas [43]	2013	76 PKU patients	33 female, 43 male	51 children, 13 adolescents, 12 young adults (<21 years)	4.c	Case series
Jurecki et al. [46]	2017	3,772 PKU patients	Not specified	41% adults	4.b	Cross-sectional study
Mlčoch, Puda, Ješina, Lhotáková, Štěrbová, and Doležal [49]	2018	184 PKU patients	89 Female, 95 Male	Mean age of PKU patients = 14.0 years (median 11; IQR 5–22)	4.b	Cross-sectional study
Teruya, Remor, and Schwartz [53]	2020	23 PKU patients	9 female, 14 male	Ages: 6–34 years; mean age = 18.0 ± 7.3 years	4.b	Cross-sectional study
Kenneson and Singh [54]	2021	219 PKU patients	78 adults (53 females, 25 males); 141 children (71 females, 70 males)	Adults: 18–52 years (median = 30 years); children: 0–22 years (median = 7.0 years)	3.e	Observational study without a control group
Teruya, Remor, and Schwartz [55]	2021	29 PKU patients	14 female, 15 male	Ages: 6–34 years (mean 16.4 ± 7.5 years)	3.e	Observational study without a control group
Rovelli et al. [58]	2021	192 PKU patients	91 female, 101 male	Mean age = 21.9 years (age range 4–65 years)	3.e	Observational study without a control group
Walkowiak et al. [59]	2021	567 PKU patients	296 female, 275 male	Age range: 0.17–53 years, Mean age = 14.77 ± 12.6 years	4.b	Cross-sectional study
Peres et al. [60]	2021	55 PKU patients	30 female, 25 male	Ages: 23.3 ± 4.3 years	3.e	Observational study without a control group
Schoen and Singh [62]	2022	28 females with PKU	28 female	Ages: 15–22 years	3.c	Cohort study with control group
Caregivers/Parents						
Bekhof et al. [27]	2003	223 (161 parents + 62 patients)	Not specified	Parents of patients’ ages: 1–22 years; Patients aged 12–22 years	3.c	Cohort study with control group
Crone et al. [30]	2005	167 parents of PKU patients	Not specified	Parents of PKU patients born between 1974 and 1995	4.b	Cross-sectional study
Ievers-Landis et al. [31]	2005	30 (19 caregivers + 11 children)	Not specified	Parents of patients’ ages: 1 to 20 years	4.b	Cross-sectional study
Ozel, Kucukkasap, Koksal, Sivri, Dursun, Tokatli, and Coskun [36]	2008	144 PKU patients	63 female, 81 male	Ages: 1–15 years	3.e	Observational study without a control group
Medford, Hare, Carpenter, Rust, Jones, and Wittkowski [45]	2017	46 maternal carers of PKU patients	45 female, 1 male	Ages: 22 to 66 years	4.b	Cross-sectional study
Borghi, Salvatici, Banderali, Riva, Giovannini, and Vegni [56]	2021	91 families of PKU patients	84 mothers, 54 fathers	Mean age: 40.8 years (SD = 7, range 23–63 years)	4.b	Cross-sectional study
Zamani, Karimi-Shahanjarini, Tapak, and Moeini [57]	2021	44 parents/caregivers of PKU children	23 male, 21 female	Parents of patients’ ages: 1–12 years	1.c	RCT

**Table 2:** An overview of studies investigating the influence of family-related factors on dietary compliance in PKU.

Study	Participants	Factors	Main Findings
Social factors			
Fehrenbach and Peterson [16]	Children and adolescents (<18 years)	Family structure	Compliant families had more structure and rules than noncompliant ones (high socioeconomic status: M = 55.63 vs. 44.25; low socioeconomic status: M = 57.87 vs. 52.00).
Olsson et al. [34]	Children and adolescents (<18 years)	Parents’ marital status	Children with separated or divorced parents had higher levels, even after adjusting for confounders.
Vieira et al. [44]	Children and adolescents (<18 years)	Living with parents	Living with both parents was protective for adherence (RR 0.59, 95% CI 0.39–0.80, *p* = 0.001).
Alaei et al. [41]	Mixed age groups	Divorced and unemployed parents; number of affected children	Blood Phe levels were higher in children with divorced (*p* = 0.02) or unemployed parents (*p* = 0.03) and positively correlated with the number of affected children per family (r = 0.43, *p* < 0.001). Parental education, family size, and diet adherence showed no significant link.
Crone et al. [30]	Caregivers/Parents	Parental attitudes and subjective norm Country of birth of the mother	Children’s Phe levels are lower (−103 µmol/L) when parents believe that their dietary adherence is good. Conversely, Phe levels increase (+156 µmol/L) when parents perceive relatives’ disapproval of dietary deviations and find it difficult to administer a synthetic protein substitute three times daily. The mother’s country of birth (native vs. foreign) also affects children’s Phe levels. Children of foreign-born mothers tend to have higher Phe concentrations, potentially due to language barriers, cultural differences in diet, and varying levels of adherence to medical instructions.
Psychological and behavioral factors			
Fehrenbach and Peterson [16]	Children and adolescents (<18 years)	Parents’ problem-solving skills	Parents of children in good dietary control produced a higher quality verbal response (M = 1.99, SD = 0.24) than noncompliant parents.
Antshel et al. [29]	Children and adolescents (<18 years)	Child and parent attributions	Phe levels significantly correlate with the external attribution of behavioral dysregulation (r = 0.69, *p* < 0.001) and academic difficulties (r = 0.52, *p* < 0.001).
Ievers-Landis et al. [31]	Caregivers/Parents	Parenting strategies, Frequency difficulty affective intensity and number of formula-related and total problems	Caregivers who rated dietary problems as less frequent, difficult, and emotionally upsetting and strategies as more effective for solving problems had children with significantly lower Phe levels (all *p* values < 0.05).
Medford et al. [45]	Caregivers/Parents	Perceived support from family, parental wellbeing	The inclusion of perceived support from family, parental wellbeing, and the level of child dependency in the regression model increased the percentage of variance explained from 14.7 to 34.1%.
Borghi et al. [56]	Caregivers/Parents	Parents’ mental wellbeing	Optimal adherence to the diet was associated with parental low social functioning, a higher tendency to control anger expression, and greater somatic depressive symptoms.
Educational factors			
Shulman et al. [17]	Children and adolescents (<18 years)	Parental education levels	Higher parent education levels were associated with lower blood Phe levels, with significant correlations found between blood Phe and maternal education (r = −0.27, *p* < 0.05) and paternal education (r = −0.28, *p* < 0.05).
Gleason et al. [18]	Children and adolescents (<18 years)	Parental knowledge	Subjects who successfully completed the program had a lower baseline blood Phe concentration (13.4 mg/dL) compared to nonsuccessful subjects (17.9 mg/dL, *p* < 0.05).
Olsson et al. [34]	Children and adolescents (<18 years)	Parental education levels	Statistical analysis showed no significant link between parents’ education and Phe levels.
Vieira et al. [44]	Children and adolescents (<18 years)	Parental education levels	Patients whose mothers had 4 years of formal education or less were at greater risk of non-adherence (RR 1.59, 95% CI 1.01–2.51, *p* = 0.044).
Cotugno et al. [40]	Mixed age groups	Parental education levels	Children with mothers with lower educational levels showed significant excess (median 158 mg vs. −36 mg, *p* = 0.045), while no difference was found based on fathers’ education (median −35.5 vs. −2.00, *p* = 0.9).
Bekhof et al. [27]	Caregivers/Parents	Parental knowledge	Linear regression analysis showed that greater knowledge was associated with lower Phe concentrations, but this association was statistically significant only in the parent group (parents: b = −28, 95% CI −39/−17; patients: b = −20, 95% CI −42/2).
Ozel et al. [36]	Caregivers/Parents	Maternal knowledge	Better maternal understanding of Phe exchange correlated with lower blood Phe in children (*p* < 0.05), indicating improved dietary compliance.

**Table 3:** An overview of studies investigating the influence of patient-specific factors on dietary compliance in PKU.

Study	Participants	Factors	Main Findings
Psychological and behavioral factors			
Singh, Kable, Guerrero, Sullivan, and Elsas [24]	Children and adolescents (<18 years)	Knowledge of diet, attitudes toward disease, health beliefs	Increased knowledge, improved attitudes, and reduced negative health beliefs were not accompanied with sustained reductions in blood Phe levels.
Antshel, Brewster, and Waisbren [29]	Children and adolescents (<18 years)	Child and parent attributions	Four vignettes compared attributional style of PKU children and their parents to children with other chronic conditions and healthy children. Children with high Phe levels attribute behavioral dysregulation to external causes (r = 0.61, *p* < 0.001), consistent across vignettes (r = 0.43, *p* < 0.001), linking Phe levels to externalizing problems. Phe levels correlate with external attribution of behavioral dysregulation (r = 0.69, *p* < 0.001) and academic difficulties (r = 0.52, *p* < 0.001).
Waisbren, Hamilton, St James, Shiloh, and Levy [20]	Adults (>18 years)	Need for support from parents, support from health professionals, external health locus of control (chance)	Logistic regression showed that women with PKU are at higher risk for poor metabolic control lacked professional support, needed substantial parental help, and believed in chance or external locus of control (a perspective where individuals view external influences as the main drivers of their life experiences and outcomes, rather than their own actions or decisions).
Brown et al. [25]	Adults (>18 years)	Believing that the costs of treatment add to the complications of the diet	The main findings indicated that late metabolic control is significantly associated with the belief that treatment costs exacerbate dietary complications, which has an RR of 4.2.
Riva, Madotto, Turato, Salvatici, and Indovina [47]	Adults (>18 years)	Work schedule	Full-time employment and shift work are associated with higher mean Phe levels, exceeding 281.11 μMol/L and 356.73 μMol/L, respectively, indicating poorer metabolic compliance.
Teruya, Remor, and Schwartz [53]	Mixed age groups	Perceived barriers related to PKU treatment.	Adolescents reported significantly fewer treatment barriers than adults (*p* = 0.008). Patients with better metabolic control perceived fewer barriers than those with poor adherence (*p* = 0.009). More perceived barriers correlated with higher Phe levels (*p* = 0.001).
Ievers-Landis et al. [31]	Caregivers/Parents	Parenting strategies, Frequency difficulty affective intensity and number of formula-related and total problems, Adherence strategies	Caregivers who perceive fewer dietary problems and find their strategies effective have children with lower Phe levels (*p* < 0.05). Authoritarian parenting correlates with higher Phe in older children (*p* < 0.05). Formula-related (r = 0.51, *p* = 0.03) and total problems (r = 0.47, *p* = 0.05) positively correlate with Phe levels. Perceived management effectiveness strongly correlates with lower Phe (r = 0.64, *p* < 0.01), while children’s maladaptive strategies are associated with higher levels, highlighting the impacts of problem-solving and parenting on adherence.
Educational factors			
Gleason, Michals, Matalon, Langenberg, and Kamath [18]	Children and adolescents (<18 years)	Patients’ PKU knowledge	Seven subjects (5M, 2F) succeeded; nine (4M, 5F) did not succeed. Groups differed only in baseline blood Phe (successful: 13.4 mg/dL; nonsuccessful: 17.9 mg/dL, *p* < 0.05). Successful subjects reduced levels by 2.9 mg/dL to 10.5 ± 3.8 mg/dL. Baseline levels negatively correlated with success (r = −0.485).
Brown et al. [25]	Adults (>18 years)	Patient’s education	Late metabolic control is significantly associated with having a high school education or less, which has an RR of 8.4.
Iakovou and Schulpis [50]	Adults (>18 years)	Patient’s education	Higher education predicted better adherence to the Phenylketonuria (PKU) diet.
Firman, Ramachandran, and Whelan [64]	Adults (>18 years)	Patients’ PKU knowledge	Knowledge levels (general, PKU-specific, and PKU diet) were significantly associated with PKU diet adherence. Those not following their PKU diet had lower knowledge scores (69.1% ± 15.4%) than those consistently adhering (78.0% ± 12.0%, *p* = 0.005).
Durham-Shearer, Judd, Whelan, and Thomas [35]	Mixed age groups	Patients’ PKU knowledge	This study found a significant improvement in knowledge scores from baseline to 1 month favoring the intervention group (*p* < 0.05), but this increase in knowledge did not lead to a statistically significant improvement in compliance measures.
Demographic factors			
Al-Qadreh et al. [23]	Children and adolescents (<18 years)	Patients’ age and sex	Phe levels were higher in patients over 8 years old (21/26) than in younger children (3/22). Age was positively correlated with Phe concentration (r = 0.60, *p* < 0.001), while BD and the artificial-to-natural-protein-intake ratio showed negative correlations (r = −0.56 and r = −0.46, respectively; *p* < 0.001).
VanZutphen et al. [33]	Children and adolescents (<18 years)	Patients’ age	Age was inversely correlated with dietary adherence, indicating lower adherence in older individuals (r = 2.53, *p* < 0.05). Both concurrent and mean lifetime Phe (Phe) levels inversely correlated with adherence (mean lifetime Phe: r = 2.60, *p* < 0.05; concurrent Phe: r = 2.82, *p* < 0.001. Concurrent Phe levels positively correlated with age (r = 0.60, *p* < 0.05)
Olsson, Montgomery, and Alm [34]	Children and adolescents (<18 years)	Patients’ age and sex	Girls tended to have lower levels than boys, with borderline statistical significance.
García et al. [48]	Children and adolescents (<18 years)	Patients’ age	Mean blood Phe (Phe) levels significantly increased in the first three years of life and between 6 and 9 years old, with a notable rise of 1.1 mg/dL from the first to the second year. Higher Phe levels at 12–23 months (OR = 1.751) and 6–8 years (OR = 1.682) were significant predictors of treatment discontinuation before age 13, explaining 29–30% of the variance in adherence.
Brown et al. [25]	Adults (>18 years)	Patients’ age	The main findings indicate that late metabolic control is significantly associated with being under 25 years of age, with a relative risk (RR) of 8.4.
Riva, Madotto, Turato, Salvatici, and Indovina [47]	Adults (>18 years)	Patients’ age	Age and employment status were found to impact Phe (Phe) blood levels. Each year of age increased mean Phe levels by 30.56 μMol/L (95% CI: 7.53; 53.60).
McMurry, Chan, Leonard, and Ernst [19]	Mixed age groups	Patients’ age	Older subjects had higher Phe levels than younger groups. Only 2/26 PKU subjects had optimal levels (<300 μmol/L), while 19 had elevated levels (>600 μmol/L). Age was correlated with Phe concentration (r = 0.636, *p* < 0.001) and negatively correlated with dietary compliance (r = −0.689, *p* < 0.0001). Older subjects’ formula compliance varied (0–100%).
Schulz and Bremer [21]	Mixed age groups	Patients’ age	Mean plasma Phe levels of the PKU patients increased with age (r = 0.35, *p* < 0.001).
Viau et al. [39]	Mixed age groups	Patients’ age	Blood Phe (Phe) levels increased with age, while Phe sampling frequency decreased. Significant differences were found in Phe level consistency and mean SD among age groups (*p* < 0.05), with marked differences between 0 and 5 years old and both 6 and 10 and >10 years old (*p* < 0.001). No significant difference in SD blood Phe levels was seen between 0 and 5 and >10 years old (*p* = 0.932).
Cotugno, Nicolò, Cappelletti, Goffredo, Dionisi Vici, and Di Ciommo [40]	Mixed age groups	Patients’ age and gender	Age (q = 0.03, *p* = 0.8) and gender did not significantly affect Phe levels (median −35.5 vs. −2.00, *p* = 0.9).
Macdonald, Nanuwa, Parkes, Nathan, and Chauhan [42]	Mixed age groups	Patients’ sex	Females had higher mean proportions, with 70%+ Phe concentrations within target range vs. males: 0-5 years; Males were more responsive (nearly significant) for OR 0.42 [0.17, 1.03], 6-17 years: OR 1.67 [1.09, 2.55], and 18+ years: OR 3.92 [1.16, 13.32].
Freehauf, Van Hove, Gao, Bernstein, and Thomas [43]	Mixed age groups	Patients’ age and sex	Phe (Phe) levels were correlated significantly with age, increasing from adolescence to young adulthood (PheMedian ρ = 0.62), particularly in adolescents and adults (Spearman PheMedian ρ = 0.48, *p* = 0.014).
Jurecki et al. [46]	Mixed age groups	Patients’ age	Non-adherence to target Phe levels increased with age.
Mlčoch, Puda, Ješina, Lhotáková, Štěrbová, and Doležal [49]	Mixed age groups	Patients’ age	Age was the strongest predictor of Phe (Phe) levels. A linear regression model revealed a starting Phe concentration of 140 μmol/L, with an increase of 22 μmol/L per year of age (*p* < 0.0001).
Kenneson and Singh [54]	Mixed age groups	Patients’ age and sex	Individuals with elevated Phe levels were mostly older females. In total, 39.5% of females vs. 21.5% of males had high Phe (*p* = 0.0202). Females were older (median 16.6 years) than males (median 9.6 years, *p* = 0.0088). No significant links were found between Phe levels and other factors studied.
Medford, Hare, Carpenter, Rust, Jones, and Wittkowski [45]	Caregivers/Parents	Patients’ age	Age significantly influenced target blood Phe levels, explaining 14.7% (*p* = 0.009) and a slightly smaller but significant portion of the variance, respectively (*p* = 0.002)

**Table 4:** An overview of studies investigating the influence of environmental factors on dietary compliance in PKU.

Study	Participants	Factors	Main Findings
Environmental factors			
Singh et al. [24]	Children and adolescents (<18 years)	Knowledge of diet	Increased knowledge was not accompanied with sustained reductions in blood Phe levels.
Peipert et al. [38]	Children and adolescents (<18 years)	Camp-based intervention	Educational and camp-based interventions have been shown to improve adherence to a low-Phe diet in patients with PKU.
Vieira et al. [44]	Children and adolescents (<18 years)	Distance to clinic	The distance between the patient’s town of origin and the PKU clinic did not correlate with median Phe concentration in the 12 months prior to study inclusion, both for adherent patients (*p* = 0.629) and non-adherent patients (*p* = 0.72).
Becsei et al. (2022) [63]	Children and adolescents (<18 years)	COVID-19 pandemic	During the study, both age groups had significantly increased median Phe (Phe) levels and fewer patients achieved target Phe ranges. Significant negative correlations were found between the dried blood spot (DBS) testing frequencies and Phe levels in both age groups during NCE (children r = −0.43, *p* = 0.002; adolescents r = −0.37, *p* = 0.012), as well as in the adolescent group during CE (r = −0.6, *p* = 0.006).
Waisbren et al. (1995) [20]	Adults (>18 years)	Support from health professionals	Logistic regression showed women at higher risk of poor metabolic control lacked professional support.
Iakovou and Schulpis [50]	Adults (>18 years)	Psychological support	All the participants who were either mildly or moderately depressed experienced a beneficial effect on their depression when they were psychologically supported, which was further enforced by the reduction in Phe blood levels after their psychological support.
Waisbren et al. (1997) [22]	Mixed age groups	Camp-based intervention	The week-long camping experiment was offered to girls and young women throughout the United States and other countries. Blood Phe levels decreased in 96% (24/25) of participants during camp and remained lower in 75% (18/24) at follow-up (*p* < 0.05).
Freehauf et al. [43]	Mixed age groups	Distance to clinic	No significant correlation was found between the distance to clinic in miles and the Phe target (*p* = 0.62).
Jurecki et al. [46]	Mixed age groups	Clinic staffing resources	Staffing resources, defined as the number of specialists per 100 actively managed PKU patients, were correlated with the proportion of non-adherent patients in different age groups.
Rovelli et al. [58]	Mixed age groups, patients ≥4 years old in follow-up, divided into age subgroups: GROUP A (n = 51, <12 years old) and GROUP B (n = 141, ≥12 years old)	COVID-19 pandemic	Children from Group A showed no significant change, while adolescents and adults from Group B improved, with blood Phe levels decreasing from 556.4 ± 301 µmol/l to 454 ± 252 µmol/L.
Walkowiak et al. [59]	Mixed age groups	COVID-19 pandemic, stress	High stress was linked to elevated Phe (*p* = 0.0023). Better compliance was associated with accepting remote consultations, fewer issues contacting providers, and a lower likelihood of skipping Phe tests.
Schoen and Singh (2022) [62]	Mixed age groups	Camp-based intervention	After the camp intervention, plasma Phe concentrations decreased significantly (median change: −173 µmol/L [IQR: −325, −28 µmol/L]), and 70% of participants with PKU showed improved dietary adherence through reduced Phe intake or increased medical food consumption.
Zamani et al. [57]	Caregivers/Parents	Educational intervention	An educational intervention significantly improved children’s Phe levels over time (F = 4.68, *p* = 0.03). From baseline to 24-month follow-up, children with normal levels increased from 26% to 73.9% in the educational group.

**Table 5:** An overview of studies investigating the influence of therapy-related factors on dietary compliance in PKU.

Study	Participants	Factors	Main Findings
Therapy-related factors			
Walkowiak et al. (2019) [51]	Children and adolescents (<18 years)	Number of visits to a specialist, number of Phe blood tests	In the first year of life, PKU patients had more doctor visits (OR = 6.8267; *p* < 0.0001) and blood tests (OR = 2.7875; *p* < 0.0402) than in the second year and beyond. More specialist visits correlated with higher Phe levels (ρ = 0.39), while more Phe blood tests correlated with better dietary control (ρ = −0.33).
Zubarioglu et al. (2022) [61]	Children and adolescents (<18 years)	Telemedicine	During the pandemic, an online monitoring system significantly improved the maintenance of Phe levels within recommended ranges for all treatment modalities. Phe washout frequency decreased, and Phe tolerance increased significantly among those on a low-Phe diet, highlighting the pandemic’s unexpected positive impact on Phe-level management.
MacDonald et al. (2006) [32]	Adults (>18 years)	Liquid protein substitute	Using a liquid protein substitute reduced self-consciousness and improved portability, making it easier to consume away from home. Significant improvements were seen in ease of use (*p* < 0.0001), convenience (*p* = 0.002), and reduced wastage (*p* = 0.001).
Burlina et al. (2020) [52]	Adults (>18 years)	Large neutral amino acid supplements	Pre-treatment, 25% reported medium and most reported low medication adherence, but 60% claimed full adherence in the last month. Post-LNAA treatment, 96% achieved full adherence. Phe levels remained stable, while Tyr levels rose significantly in 92% of patients (mean 75 ± 16 µmol/L; *p* = 0.0195).
MacDonald et al. (2003) [26]	Mixed age groups	Phe-free amino acid tablets vs. powder	Compliance was higher with the new tablets compared to usual protein substitutes, with 90% adherence versus 65%. Furthermore, plasma Phe levels were lower with the amino acid tablets, showing a median difference of 46 µmol/L in blood concentrations between the groups, which was statistically significant (*p* = 0.02).
Camfield et al. (2004) [28]	Mixed age groups	Treatment approach	Children aged 2–12 in Nova Scotia had better Phe control and more medical consultations than those in New Brunswick (*p* < 0.01). Older patients had more frequent elevated Phe levels (*p* = 0.01), indicating that expert, coordinated PKU management is more effective.
Jurecki et al. (2017) [46]	Mixed age groups	Patient age, clinic staffing resources, clinic Phe target, adherence to blood frequency testing	Non-adherence to target Phe levels increased with age. Clinics using a higher upper Phe target (600 μM) for adults showed more non-adherence compared to those with a 360 μM target (*p* < 0.05). Adherence to target Phe levels did not correlate with adherence to or actual frequency of blood testing, except for patients who are pregnant/planning pregnancy. Staffing resources correlated with the proportion of non-adherent patients in different age groups.
Teruya et al. (2021) [55]	Mixed age groups	Tolerance to Phe, metabolic control throughout childhood, perceived difficulty in living with demands of treatment	Classical PKU patients had significantly higher current blood Phe levels than mild PKU patients (U = 37.000, *p* = 0.003). Lifetime and childhood Phe levels strongly correlated with recent metabolic control. More perceived treatment barriers were associated with higher blood Phe levels (τ = 0.39, *p* = 0.003).
Peres et al. (2021) [60]	Mixed age groups	Transition program from pediatric to adult services	Median blood Phe levels stayed stable (525 ± 248 µmol/L vs. 552 ± 225 µmol/L; *p* = 0.100), but the percentage below 480 µmol/L decreased (51% to 37%; *p* = 0.041). Clinic appointments increased significantly (from 5 to 11; *p* < 0.001). The adult service minimally affected metabolic control but maintained high attendance.

## Data Availability

Not applicable.

## Supplementary Materials

The following supporting information can be downloaded at https://www.mdpi.com/article/10.3390/nu16183119/s1, Table S1: Search terms per database, Table S2: QuADS item scores per study.
