# Supplementary material for: Factors Affecting Adherence to a Low Phenylalanine Diet in Patients with Phenylketonuria: A Systematic Review
Source: Nutrients. 2024 Sep 15;16(18):3119. doi: 10.3390/nu16183119 (PMC11435312; doi:10.3390/nu16183119)
Supplement: Supplementary file 1 [file nutrients-16-03119-s001.zip › nutrients-3132702-supplementary.pdf]

Table S1. Search terms per database.

Databases	Search terms
PubMed	("phenylketonurias"[MeSH Terms] OR "phenylketonurias"[All Fields] OR "phenylketonuria"[All Fields] OR ("PKU"[All Fields] OR ("hyperphenylalaninemias"[All Fields] OR "phenylketonurias"[MeSH Terms] OR "phenylketonurias"[All Fields] OR "hyperphenylalaninaemia"[All Fields] OR "hyperphenylalaninaemias"[All Fields] OR "hyperphenylalaninemia"[All Fields] OR ("hyperphenylalaninemias"[All Fields] OR "phenylketonurias"[MeSH Terms] OR "phenylketonurias"[All Fields] OR "hyperphenylalaninaemia"[All Fields] OR "hyperphenylalaninaemias"[All Fields] OR "hyperphenylalaninemia"[All Fields] OR ((("phenylalanin"[All Fields] OR "phenylalanine"[Supplementary Concept] OR "phenylalanine"[All Fields] OR "phenylalanine"[MeSH Terms] OR "phenylalanines"[All Fields]) AND (("mixed function oxygenases"[Supplementary Concept] OR "mixed function oxygenases"[All Fields] OR "hydroxylase"[All Fields] OR "mixed function oxygenases"[MeSH Terms] OR ("mixed"[All Fields] AND "function"[All Fields] AND "oxygenases"[All Fields]) OR "hydroxylases"[All Fields]) AND ("deficiences"[All Fields] OR "deficiencies"[All Fields] OR "deficiency"[MeSH Subheading] OR "deficiency"[All Fields] OR "deficient"[All Fields] OR "deficients"[All Fields]))) OR ("PAH"[All Fields] AND ("deficiences"[All Fields] OR "deficiencies"[All Fields] OR "deficiency"[MeSH Subheading] OR "deficiency"[All Fields] OR "deficient"[All Fields] OR "deficients"[All Fields]))))))) AND (("adherance"[All Fields] OR "adhere"[All Fields] OR "adhered"[All Fields] OR "adherence"[All Fields] OR "adherences"[All Fields] OR "adherent"[All Fields] OR "adherents"[All Fields] OR "adherer"[All Fields] OR "adherers"[All Fields] OR "adheres"[All Fields] OR "adhering"[All Fields] OR ("compliances"[All Fields] OR "patient compliance"[MeSH Terms] OR ("patient"[All Fields] AND "compliance"[All Fields]) OR "patient compliance"[All Fields] OR "compliance"[All Fields] OR "compliance"[MeSH Terms] OR ("concordance"[All Fields] OR "concordances"[All Fields] OR "concordancy"[All Fields] OR "concordant"[All Fields] OR ("cooperate"[All Fields] OR "cooperated"[All Fields] OR "cooperates"[All Fields] OR "cooperating"[All Fields] OR "cooperation"[All Fields] OR "cooperations"[All Fields] OR "cooperative"[All Fields] OR "cooperatively"[All Fields] OR "cooperatives"[All Fields] OR "cooperativities"[All Fields] OR "cooperativity"[All Fields] OR "cooperator"[All Fields] OR "cooperators"[All Fields] OR ("persist"[All Fields] OR "persistance"[All Fields] OR "persistant"[All Fields] OR "persisted"[All Fields] OR "persistence"[All Fields] OR "persistences"[All Fields] OR "persistencies"[All Fields] OR "persistency"[All Fields] OR "persistent"[All Fields] OR "persistently"[All Fields] OR "persistents"[All Fields] OR "persister"[All Fields] OR "persisters"[All Fields] OR "persisting"[All Fields] OR "persists"[All Fields]))))) AND ("diet"[MeSH Terms] OR "diet"[All Fields] OR ("diet"[MeSH Terms] OR "diet"[All Fields] OR "dietary"[All Fields] OR "dietaries"[All Fields] OR ("food"[MeSH Terms] OR "food"[All Fields] OR ("low-Phe"[All Fields] OR (("low"[All Fields] AND ("phenylalanin"[All Fields] OR "phenylalanine"[Supplementary Concept] OR "phenylalanine"[All Fields] OR "phenylalanine"[MeSH Terms] OR "phenylalanines"[All Fields])) OR ("therapeutics"[MeSH Terms] OR "therapeutics"[All Fields] OR "treatments"[All Fields] OR "therapy"[MeSH Subheading] OR "therapy"[All Fields] OR "treatment"[All Fields] OR "treatment s"[All Fields])))))))
Cochrane Library	((phenylketonuria) OR (PKU) OR (hyperphenylalaninemia) OR (hyperphenylalaninaemia) OR (phenylalanine hydroxylase deficiency) OR (PAH deficiency)) AND ((adherence) OR (compliance) OR (concordance) OR (cooperation) OR (persistence)) AND ((diet) OR (dietary) OR (food) OR (low-Phe) OR (low

	phenylalanine) OR (treatment)) in Title Abstract Keyword - (Word variations have been searched)
lens.org	(Title: (((phenylketonuria) OR ((PKU) OR ((hyperphenylalaninemia) OR ((hyperphenylalaninaemia) OR ((phenylalanine AND (hydroxylase AND deficiency)) OR (PAH AND deficiency)))))) AND (((adherence) OR ((compliance) OR ((concordance) OR ((cooperation) OR (persistence))))) AND ((diet) OR ((dietary) OR ((food) OR ((low-Phe) OR ((low AND phenylalanine) OR (treatment)))))))) OR (Abstract: (((phenylketonuria) OR ((PKU) OR ((hyperphenylalaninemia) OR ((hyperphenylalaninaemia) OR ((phenylalanine AND (hydroxylase AND deficiency)) OR (PAH AND deficiency)))))) AND (((adherence) OR ((compliance) OR ((concordance) OR ((cooperation) OR (persistence))))) AND ((diet) OR ((dietary) OR ((food) OR ((low-Phe) OR ((low AND phenylalanine) OR (treatment)))))))) OR (Keyword: (((phenylketonuria) OR ((PKU) OR ((hyperphenylalaninemia) OR ((hyperphenylalaninaemia) OR ((phenylalanine AND (hydroxylase AND deficiency)) OR (PAH AND deficiency)))))) AND (((adherence) OR ((compliance) OR ((concordance) OR ((cooperation) OR (persistence))))) AND ((diet) OR ((dietary) OR ((food) OR ((low-Phe) OR ((low AND phenylalanine) OR (treatment)))))))) OR Field of Study: (((phenylketonuria) OR ((PKU) OR ((hyperphenylalaninemia) OR ((hyperphenylalaninaemia) OR ((phenylalanine AND (hydroxylase AND deficiency)) OR (PAH AND deficiency)))))) AND (((adherence) OR ((compliance) OR ((concordance) OR ((cooperation) OR (persistence))))) AND ((diet) OR ((dietary) OR ((food) OR ((low-Phe) OR ((low AND phenylalanine) OR (treatment)))))))))))
dimensions.ai	((phenylketonuria) OR (PKU) OR (hyperphenylalaninemia) OR (hyperphenylalaninaemia) OR (HPA) OR (phenylalanine hydroxylase deficiency) OR (PAH deficiency)) AND ((adherence) OR (compliance) OR (concordance) OR (cooperation) OR (persistence)) AND ((diet) OR (dietary) OR (food) OR (nutrient) OR (low-Phe) OR (low phenylalanine)) - in Title and Abstract1

Table S2. QuADS item scores per study.

Study number	QuADS item													Total score
	1.	2	3	4	5	6	7	8	9	10	11	12	13	
1[16]	3	3	3	3	1	1	2	2	1	2	2	1	2	26
2[17]	2	2	3	3	1	1	2	2	1	2	2	1	1	23
3[18]	2	3	2	2	1	1	2	2	1	2	2	1	2	23
4[19]	2	3	3	3	1	2	3	3	2	3	3	1	1	30
5[20]	1	2	2	2	1	2	2	2	1	1	2	1	2	21
6[21]	1	2	2	2	1	2	2	2	1	1	2	1	2	21
7[22]	2	3	3	3	2	2	2	3	2	2	3	2	2	31
8[23]	3	2	3	3	1	2	3	3	1	2	3	1	1	28
9[24]	3	3	3	3	1	3	3	3	1	3	3	1	1	31
10[25]	2	3	3	3	1	2	3	2	2	2	3	1	2	29
11[26]	2	3	2	3	1	1	2	2	1	1	3	0	2	23
12[27]	2	3	3	3	2	2	2	3	2	2	3	2	2	31
13[28]	3	2	3	2	1	2	2	3	3	2	3	1	2	29
14[29]	2	2	3	2	1	2	2	3	3	1	3	1	2	27
15[30]	2	3	3	3	1	2	2	3	3	1	2	1	3	29
16[31]	2	3	3	3	2	1	3	2	2	1	3	0	2	27
17[32]	2	3	3	3	1	2	3	2	2	2	3	1	1	28

18[33]	3	2	3	3	1	2	2	2	3	3	3	1	2	30
19[34]	3	3	3	3	1	2	3	3	2	2	3	2	2	32
20[35]	1	2	3	2	1	1	2	2	1	2	2	0	1	20
21[36]	3	3	2	2	2	2	3	2	2	2	2	1	2	28
22[37]	2	2	2	3	2	2	2	1	2	1	2	1	2	24
23[38]	3	3	3	3	2	3	3	3	1	3	3	1	3	34
24[39]	3	2	3	3	1	1	3	3	0	1	3	0	0	23
25[40]	1	1	3	2	1	1	2	2	2	1	2	0	0	18
26[41]	3	3	3	3	2	3	3	3	1	3	3	1	3	34
27[42]	3	2	3	3	1	2	3	3	2	3	3	0	2	30
28[43]	2	3	3	3	1	2	2	3	2	2	2	1	2	28
29[44]	2	3	3	2	2	3	3	3	2	2	2	1	2	30
30[45]	2	3	3	2	2	2	3	3	2	1	2	1	2	28
31[46]	2	3	3	2	1	1	2	2	2	1	2	1	2	24
32[47]	3	2	3	3	2	2	3	2	2	2	3	2	2	31
33[48]	2	3	3	3	2	3	3	3	2	1	2	2	1	30
34[49]	2	3	2	2	1	2	2	2	2	2	2	1	1	24
35[50]	3	2	3	3	2	2	2	3	3	2	3	1	2	31
36[51]	2	3	3	2	1	1	2	2	3	2	3	1	2	27
37[52]	2	3	3	3	2	2	2	3	3	2	2	1	2	30
38[53]	2	3	2	2	1	2	2	2	2	2	2	1	2	25
39[54]	2	2	3	3	2	3	3	3	3	1	2	1	1	29
40[55]	3	3	3	3	2	2	3	3	3	2	3	1	2	33
41[56]	2	3	3	3	1	2	3	2	2	3	3	2	2	31
42[57]	2	3	2	3	2	2	3	1	1	1	2	1	1	24
43[58]	2	3	3	2	2	2	2	3	2	2	2	1	2	28
44[59]	2	3	3	3	2	2	2	3	3	2	3	2	2	32
45[60]	3	3	3	3	1	2	2	3	2	1	3	1	2	29
46[61]	2	3	2	3	2	2	3	3	2	2	2	1	2	29
47[62]	2	2	3	2	2	2	2	3	1	2	2	1	2	26
48[63]	3	3	2	3	2	3	3	3	2	2	2	2	2	32
49[64]	2	3	2	3	2	2	3	2	2	1	3	2	2	29

QuADS Items and Scoring Criteria Descriptions [15]

1. Theoretical or conceptual underpinning to the research

0 - No mention at all.

1 - General reference to broad theories or concepts that frame the study. e.g. key concepts were identified in the introduction section.

2 - Identification of specific theories or concepts that frame the study and how these informed the work undertaken. e.g. key concepts were identified in the introduction section and applied to the study.

3 - Explicit discussion of the theories or concepts that inform the study, with application of the theory or concept evident through the design, materials and outcomes explored. e.g. key concepts were identified in the introduction section and the application apparent in each element of the study design.

2. Statement of research aim/s

0 - No mention at all.

1 - Reference to what the sought to achieve embedded within the report but no explicit aims statement.

2 - Aims statement made but may only appear in the abstract or be lacking detail.

3 - Explicit and detailed statement ofaim/s in the main body of report.

3. Clear description of research setting and target population

0 - No mention at all.
1 - General description of research area but not of the specific research environment e.g. 'in primary care.'
2 - Description of research setting is made but is lacking detail e.g. 'in primary care practices in region [x]'.
3 - Specific description of the research setting and target population of study e.g. 'nurses and doctors from GP practices in [x] part of [x] city in [x] country.'

4. The study design is appropriate to address the stated research aim/s
0 - No research aim/s stated or the design is entirely unsuitable e.g. a Y/N item survey for a study seeking to undertake exploratory work of lived experiences.
1 - The study design can only address some aspects of the stated research aim/s e.g. use of focus groups to capture data regarding the frequency and experience of a disease.
2 - The study design can address the stated research aim/s but there is a more suitable alternative that could have been used or used in addition e.g. addition of a qualitative or quantitative component could strengthen the design.
3 - The study design selected appears to be the most suitable approach to attempt to answer the stated research aim/s.

5. Appropriate sampling to address the research aim/s
0 - No mention of the sampling approach.
1 - Evidence of consideration of the sample required e.g. the sample characteristics are described and appear appropriate to address the research aim/s.
2 - Evidence of consideration of sample required to address the aim. e.g. the sample characteristics are described with reference to the aim/s.
3 - Detailed evidence of consideration of the sample required to address the research aim/s. e.g. sample size calculation or discussion of an iterative sampling process with reference to the research aims or the case selected for study.

6. Rationale for choice of data collection tool/s
0 - No mention of rationale for data collection tool used.
1 - Very limited explanation for choice of data collection tool/s. e.g. based on availability of tool.
2 - Basic explanation of rationale for choice of data collection tool/s. e.g. based on use in a prior similar study.
3 - Detailed explanation of rationale for choice of data collection tool/s. e.g. relevance to the study aim/s, codesigned with the target population or assessments of tool quality.

7. The format and content of data collection tool is appropriate to address the stated research aim/s
0 - No research aim/s stated and/or data collection tool not detailed.
1 - Structure and/or content of tool/s suitable to address some aspects of the research aim/s or to address the aim/s superficially e.g. single item response that is very general or an open-response item to capture content which requires probing.
2 - Structure and/or content of tool/s allow for data to be gathered broadly addressing the stated aim/s but could benefit from refinement. e.g. the framing of survey or interview questions are too broad or focused to one element of the research aim/s.
3- Structure and content of tool/s allow for detailed data to be gathered around all relevant issues required to address the stated research aim/s.

8. Description of data collection procedure
0 - No mention of the data collection procedure.

1 - Basic and brief outline of data collection procedure e.g. ‘using a questionnaire distributed to staff’.
2 - States each stage of data collection procedure but with limited detail or states some stages in detail but omits others e.g. the recruitment process is mentioned but lacks important details.
3 - Detailed description of each stage of the data collection procedure, including when, where and how data was gathered such that the procedure could be replicated.

9. Recruitment data provided
0 - No mention of recruitment data.
1 - Minimal and basic recruitment data e.g. number of people invited who agreed to take part.
2 - Some recruitment data but not a complete account e.g. number of people who were invited and agreed.
3 - Complete data allowing for full picture of recruitment outcomes e.g. number of people approached, recruited, and who completed with attrition data explained where relevant.

10. Justification for analytic method selected
0 - No mention of the rationale for the analytic method chosen.
1 - Very limited justification for choice of analytic method selected. e.g. previous use by the research team.
2 - Basic justification for choice of analytic method selected e.g. method used in prior similar research.
3 - Detailed justification for choice of analytic method selected e.g. relevance to the study aim/s or comment around of the strengths of the method selected.

11. The method of analysis was appropriate to answer the research aim/s
0 - No mention at all.
1 - Method of analysis can only address the research aim/s basically or broadly.
2 - Method of analysis can address the research aim/s but there is a more suitable alternative that could have been used or used in addition to offer a stronger analysis.
3 - Method of analysis selected is the most suitable approach to attempt answer the research aim/s in detail e.g. for qualitative interpretative phenomenological analysis might be considered preferable for experiences vs. content analysis to elicit frequency of occurrence of events.

12. Evidence that the research stakeholders have been considered in research design or conduct.
0 - No mention at all.
Consideration of some the research stakeholders e.g. use of pilot study with target sample but no stakeholder involvement in planning stages of study design.
1 - Evidence of stakeholder input informing the research. e.g. use of pilot study with feedback influencing the study design/conduct or reference to a project reference group established to guide the research.
2 - Substantial consultation with stakeholders identifiable in planning of study design and in preliminary work e.g. consultation in the conceptualisation of the research, a project advisory group or evidence of stakeholder input informing the work.

13. Strengths and limitations critically discussed
0 - No mention at all.
1 - Very limited mention of strengths and limitations with omissions of many key issues. e.g. one or two strengths/limitations mentioned with limited detail.
2 - Discussion of some of the key strengths and weaknesses of the study but not complete. e.g. several strengths/limitations explored but with notable omissions or lack of depth of explanation.
3 - Thorough discussion of strengths and limitations of all aspects of study including design, methods, data collection tools, sample & analytic approach.

References:
15. Harrison R, Jones B, Gardner P, Lawton R. Quality assessment with diverse studies (QuADS): an appraisal tool for methodological and reporting quality in systematic reviews of mixed- or multi-method studies. BMC Health Serv Res. 2021;21(1):144.
16. Fehrenbach AM, Peterson L. Parental problem-solving skills, stress, and dietary compliance in phenylketonuria. J Consult Clin Psychol. 1989;57(2):237–41.
17. Shulman S, Fisch RO, Zempel CE, Gadish O, Chang PN. Children with phenylketonuria: the interface of family and child functioning. J Dev Behav Pediatr. 1991;12(5):315–21.
18. Gleason LA, Michals K, Matalon R, Langenberg P, Kamath S. A treatment program for adolescents with phenylketonuria. Clin Pediatr (Phila). 1992;31(6):331–5.
19. McMurry MP, Chan GM, Leonard CO, Ernst SL. Bone mineral status in children with phenylketonuria--relationship to nutritional intake and phenylalanine control. Am J Clin Nutr. 1992;55(5):997–1004.
20. Waisbren SE, Hamilton BD, St James PJ, Shiloh S, Levy HL. Psychosocial factors in maternal phenylketonuria: women's adherence to medical recommendations. Am J Public Health. 1995;85(12):1636–41.
21. Schulz B, Bremer HJ. Nutrient intake and food consumption of adolescents and young adults with phenylketonuria. Acta Paediatr. 1995;84(7):743–8.
22. Waisbren SE, Rokni H, Bailey I, Rohr F, Brown T, Warner-Rogers J. Social factors and the meaning of food in adherence to medical diets: results of a maternal phenylketonuria summer camp. J Inherit Metab Dis. 1997;20(1):21–7.
23. Al-Qadreh A, Schulpis KH, Athanasopoulou H, Mengreli C, Skarpalezou A, Voskaki I. Bone mineral status in children with phenylketonuria under treatment. Acta Paediatr. 1998;87(11):1162–6.
24. Singh RH, Kable JA, Guerrero NV, Sullivan KM, Elsas LJ. Impact of a camp experience on phenylalanine levels, knowledge, attitudes, and health beliefs relevant to nutrition management of phenylketonuria in adolescent girls. J Am Diet Assoc. 2000;100(7):797–803.
25. Brown AS, Fernhoff PM, Waisbren SE, Frazier DM, Singh R, Rohr F, Morris JM, Kenneson A, MacDonald P, Gwinn M, Honein M, Rasmussen SA. Barriers to successful dietary control among pregnant women with phenylketonuria. Genet Med. 2002;4(2):84–9.
26. MacDonald A, Ferguson C, Rylance G, Morris A, Asplin D, Hall S, Booth I. Are tablets a practical source of protein substitute in phenylketonuria? Arch Dis Child. 2003;88(4):327–9.
27. Bekhof J, van Spronsen FJ, Crone MR, van Rijn M, Oudshoorn CGM, Verkerk PH. Influence of knowledge of the disease on metabolic control in phenylketonuria. Eur J Pediatr. 2003;162(6):440–2.
28. Camfield CS, Joseph M, Hurley T, Campbell K, Sanderson S, Camfield PR. Optimal management of phenylketonuria: a centralized expert team is more successful than a decentralized model of care. J Pediatr. 2004;145(1):53–7.
29. Antshel KM, Brewster S, Waisbren SE. Child and parent attributions in chronic pediatric conditions: phenylketonuria (PKU) as an exemplar. J Child Psychol Psychiatry. 2004;45(3):622–30.
30. Crone MR, van Spronsen FJ, Oudshoorn K, Bekhof J, van Rijn G, Verkerk PH. Behavioural factors related to metabolic control in patients with phenylketonuria. J Inherit Metab Dis. 2005;28(5):627–37.
31. Ievers-Landis CE, Hoff AL, Brez C, Cancilliere MK, McConnell J, Kerr D. Situational analysis of dietary challenges of the treatment regimen for children and adolescents with phenylketonuria and their primary caregivers. J Dev Behav Pediatr. 2005;26(3):186–93.
32. MacDonald A, Lilburn M, Davies P, Evans S, Daly A, Hall SK, Hendriksz C, Chakrapani A, Lee P. "Ready to drink" protein substitute is easier is for people with phenylketonuria. J Inherit Metab Dis. 2006;29(4):526–31.
33. VanZutphen KH, Packman W, Sporri L, Needham MC, Morgan C, Weisiger K, Packman S. Executive functioning in children and adolescents with phenylketonuria. Clin Genet. 2007;72(1):13–8.
34. Olsson GM, Montgomery SM, Alm J. Family conditions and dietary control in phenylketonuria. J Inherit Metab Dis. 2007;30(5):708–15.
35. Durham-Shearer SJ, Judd PA, Whelan K, Thomas JE. Knowledge, compliance and serum phenylalanine concentrations in adolescents and adults with phenylketonuria and the effect of a patient-focused educational resource. J Hum Nutr Diet. 2008;21(5):474–85.
36. Ozel HG, Kucukkasap T, Koksal G, Sivri HSK, Dursun A, Tokatli A, Coskun T. Does maternal knowledge impact blood phenylalanine concentration in Turkish children with phenylketonuria? J Inherit Metab Dis. 2008;31 Suppl 2:S213-217.
37. Sharman R, Sullivan K, Young R, McGill J. Biochemical markers associated with executive function in adolescents with early and continuously treated phenylketonuria. Clin Genet. 2009;75(2):169–74.
38. Peipert J, Rohr F, Phornphutkul C, Waisbren S. Changes in Metabolic Control of Phenylketonuria in Children Attending a Summer Camp: Pre- and Postassessment of a Nutritional Intervention. ICAN: Infant, Child, & Adolescent Nutrition. 2010;2(2):117–9.
39. Viau KS, Wengreen HJ, Ernst SL, Cantor NL, Furtado LV, Longo N. Correlation of age-specific phenylalanine levels with intellectual outcome in patients with phenylketonuria. J Inherit Metab Dis. 2011;34(4):963–71.

40. Cotugno G, Nicolò R, Cappelletti S, Goffredo BM, Dionisi Vici C, Di Ciommo V. Adherence to diet and quality of life in patients with phenylketonuria. Acta Paediatr. 2011;100(8):1144–9.
41. Alaei M, Asadzadeh-Totonchi G, Gachkar L, Farivar S. Family Social Status and Dietary Adherence of Patients with Phenylketonuria. Iran J Pediatr. 2011;21(3):379–84.
42. Macdonald A, Nanuwa K, Parkes L, Nathan M, Chauhan D. Retrospective, observational data collection of the treatment of phenylketonuria in the UK, and associated clinical and health outcomes. Curr Med Res Opin. 2011;27(6):1211–22.
43. Freehauf C, Van Hove JLK, Gao D, Bernstein L, Thomas JA. Impact of geographic access to care on compliance and metabolic control in phenylketonuria. Mol Genet Metab. 2013;108(1):13–7.
44. Vieira TA, Nalin T, Krug BC, Bittar CM, Netto CBO, Schwartz IVD. Adherence to Treatment of Phenylketonuria: A Study in Southern Brazilian Patients. Journal of Inborn Errors of Metabolism and Screening. 2015;3:2326409815579861.
45. Medford E, Hare DJ, Carpenter K, Rust S, Jones S, Wittkowski A. Treatment Adherence and Psychological Wellbeing in Maternal Carers of Children with Phenylketonuria (PKU). JIMD Rep. 2017;37:107–14.
46. Jurecki ER, Cederbaum S, Kopesky J, Perry K, Rohr F, Sanchez-Valle A, Viau KS, Sheinin MY, Cohen-Pfeffer JL. Adherence to clinic recommendations among patients with phenylketonuria in the United States. Mol Genet Metab. 2017;120(3):190–7.
47. Riva MA, Madotto F, Turato M, Salvatici E, Indovina S, Giovannini M, Riva E, Cesana G. Work activity and phenylalanine levels in a population of young adults with classic PKU. Med Lav. 2017;108(2):118–22.
48. García MI, Araya G, Coo S, Waisbren SE, de la Parra A. Treatment adherence during childhood in individuals with phenylketonuria: Early signs of treatment discontinuation. Mol Genet Metab Rep. 2017;11:54–8.
49. Mlčoch T, Puda R, Ješina P, Lhotáková M, Štěrbová Š, Doležal T. Dietary patterns, cost and compliance with low-protein diet of phenylketonuria and other inherited metabolic diseases. Eur J Clin Nutr. 2018;72(1):87–92.
50. Iakovou K, Schulpis K. The significant role of educational status in PKU patients: the beneficial effect of psychological support in depression. Int J Adolesc Med Health. 2019;33(4).
51. Walkowiak D, Bukowska-Posadzy A, Kałużny Ł, Ołtarzewski M, Staszewski R, Musielak M, Walkowiak J. Therapy compliance in children with phenylketonuria younger than 5 years: A cohort study. Adv Clin Exp Med. 2019;28(10):1385–91.
52. Burlina AP, Cazzorla C, Massa P, Loro C, Gueraldi D, Burlina AB. The Impact of a Slow-Release Large Neutral Amino Acids Supplement on Treatment Adherence in Adult Patients with Phenylketonuria. Nutrients. 2020;12(7):2078.
53. Teruya KI, Remor E, Schwartz IVD. Development of an inventory to assess perceived barriers related to PKU treatment. J Patient Rep Outcomes. 2020;4:29.
54. Kenneson A, Singh RH. Natural history of children and adults with phenylketonuria in the NBS-PKU Connect registry. Mol Genet Metab. 2021;134(3):243–9.
55. Teruya KI, Remor E, Schwartz IVD. Factors that increase risk for poor adherence to phenylketonuria treatment in Brazilian patients. Am J Med Genet A. 2021;185(7):1991–2002.
56. Borghi L, Salvatici E, Banderali G, Riva E, Giovannini M, Vegni E. Psychological wellbeing in parents of children with phenylketonuria and association with treatment adherence. Minerva Pediatr (Torino). 2021;73(4):330–9.
57. Zamani R, Karimi-Shahanjarini A, Tapak L, Moeini B. Improving phenylalanine and micronutrients status of children with phenylketonuria: a pilot randomized study. Orphanet J Rare Dis. 2021;16(1):475.
58. Rovelli V, Zuvadelli J, Ercoli V, Montanari C, Paci S, Dionigi AR, Scopari A, Salvatici E, Cefalo G, Banderali G. PKU and COVID19: How the pandemic changed metabolic control. Mol Genet Metab Rep. 2021;27:100759.
59. Walkowiak D, Mikołuć B, Mozrzymas R, Kałużny Ł, Didycz B, Korycińska-Chaaban D, Patalan M, Jagłowska J, Chrobot A, Starostecka E, Zarębska J, Walkowiak J. The Impact of the COVID-19 Pandemic on the Perception of Health and Treatment-Related Issues among Patients with Phenylketonuria in Poland-The Results of a National Online Survey. Int J Environ Res Public Health. 2021;18(12):6399.
60. Peres M, Almeida MF, Pinto ÉJ, Carmona C, Rocha S, Guimas A, Ribeiro R, Martins E, Bandeira A, MacDonald A, Rocha JC. Implementing a Transition Program from Paediatric to Adult Services in Phenylketonuria: Results after Two Years of Follow-Up with an Adult Team. Nutrients. 2021;13(3):799.
61. Zubarioglu T, Hopurcuoglu D, Uygur E, Ahmadzada S, Oge-Enver E, Isat E, Cansever MS, Kiykim E, Aktuglu-Zeybek C. The Impact of Telemedicine for Monitoring and Treatment of Phenylketonuria Patients on Metabolic Outcome During Coronavirus Disease-19 Outbreak. Telemed J E Health. 2022;28(2):258–65.
62. Schoen MS, Singh RH. Plasma metabolomic profile changes in females with phenylketonuria following a camp intervention. Am J Clin Nutr. 2022;115(3):811–21.
63. Becsei D, Kiss E, Szatmári I, Arató A, Reusz G, Szabó AJ, Bókay J, Zsidegh P. A retrospective analysis of metabolic control in children with PKU in the COVID-19 era. Mol Genet Metab Rep. 2022;32:100897.
64. Firman SJ, Ramachandran R, Whelan K. Knowledge, perceptions and behaviours regarding dietary management of adults living with phenylketonuria. J Hum Nutr Diet. 2022;35(6):1016–29.
